# NLRP3 Inflammasome and Inflammation-Related Proteins Expression Characterized in Non-Cultivated Versus Cultivated Peripheral Blood Cells of Patients with Amyotrophic Lateral Sclerosis

**DOI:** 10.3390/neurosci7040084

**Published:** 2026-07-23

**Authors:** Denis Shevchuk, Yulia Kuzmenko, Mariya Zakharova, Vadim Karpov, Elizaveta Starodubova, Anastasia Latanova

**Affiliations:** 1 Engelhardt Institute of Molecular Biology, Russian Academy of Sciences, Vavilov Str., 32, 119991 Moscow, Russia; dvlshev@gmail.com (D.S.); kuzmenko-yulia@mail.ru (Y.K.); karpovvl2008@gmail.com (V.K.); estarodubova@yandex.ru (E.S.); 2 Russian Center of Neurology and Neurosciences, Volokolamskoye Shosse, 80, 125367 Moscow, Russia; zakharova@neurology.ru

**Keywords:** inflammation, amyotrophic lateral sclerosis, NLRP3 inflammasome, peripheral blood mononuclear cells, biomarkers, Interleukin-1β, Interleukin-18

## Abstract

Background: Cell and mouse models studies demonstrate NLRP3 inflammasome involvement in amyotrophic lateral sclerosis (ALS) neuroinflammation. Peripheral blood mononuclear cells (PBMCs) are a promising, yet understudied, source of in vivo inflammasome activation biomarkers. Our study reviewed the literature on PBMC-based inflammasome studies of ALS and other neurodegenerative diseases and tested different conditions for PBMC handling to evaluate inflammasome and inflammation-related protein expression in these cells. Methods: Expression of NLRP3 inflammasome components and inflammation-related proteins was analyzed by Real-time qPCR and Western blot in non-cultivated/cultivated PBMCs of 23 ALS patients and 20 Healthy controls. IL-1β and IL-18 levels were measured in plasma and cultivated PBMC supernatants by ELISA. Results: Cultivation of PBMCs decreased expression of inflammasome components and inflammation-related cytokines on the mRNA but not protein level. NLRP3 mRNA expression was significantly higher in ALS-cultivated PBMCs. In both ALS and Healthy controls, IL-18 was detected in plasma, and IL-1β in supernatants of cultivated PBMCs. Conclusions: Our findings suggest that PBMC handling conditions, i.e., cell cultivation, may determine particular parameters associated with NLRP3 inflammasome expression and activation pathway, so they should be carefully selected for PBMC-based studies of inflammasome in neurodegenerative and non-neurological disorders and taken into account when interpreting the study results.

## 1. Introduction

Amyotrophic lateral sclerosis (ALS) is a neurodegenerative disorder, the most common and severe form of motor neuron disease (0.3–2.5 cases per 100,000 people per year) [1]. It affects both lower and upper motor neurons, causing rapidly progressive muscle weakness and death within 3–5 years after the onset, most often due to respiratory failure. About 10% of ALS cases are familial, predominantly with an autosomal dominant inheritance, associated with mutations in SOD1, FUS, C9orf72, TARDBP and other genes, while the remaining 90% of cases are sporadic [2]. Several anti-ALS drugs are approved, but a reliable effect in extending life has been demonstrated only by riluzole, glutamatergic neurotransmission inhibitor, and it prolongs life expectancy for not more than 2–3 months [2,3,4]. The pathogenesis of ALS is determined by complex pathophysiological mechanisms including disrupted protein homeostasis leading to the formation of protein aggregates in motor neurons, astrocytes and brain parenchyma [5], oxidative stress, excitotoxicity, mitochondrial dysfunction, abnormalities in RNA processing, and neuroinflammation [1].

In ALS patients, the role of neuroinflammation in disease progression is determined in post-mortem tissue studies [6,7] and in vivo by elevated levels of soluble pro-inflammatory cytokines and chemokines detected in the blood and cerebrospinal fluid [6] and positron emission tomography (PET) imaging [8]. A well-known trigger of inflammation, including neuroinflammation, is NLRP3 inflammasome, a cytoplasmic complex present in immune and some other cell types. Typically, it consists of three proteins: a sensor protein, an adaptor protein, ASC (apoptosis-associated speck-like protein containing a caspase recruitment domain (CARD)), and an effector protein, pro-caspase-1 [9]. Several types of inflammasomes are known, which differ in the sensor protein types, the presence/absence of the ASC adaptor, and affinity for activation signals [9]. NLRP3 inflammasome, named after its NLRP3 sensor (nucleotide-binding domain, leucine-rich-containing family, pyrin domain-containing-3) is the most studied type. To be activated, NLRP3 inflammasome requires two signals. A priming signal is a substrate of PRR (pattern recognition receptor), and upon its recognition by the receptor, activates NF-κB signaling, which triggers the expression of *NLRP3* gene and *IL-1β* and *IL-18* precursors genes [10]. The second activating signal is required for the assembly of the NLRP3 inflammasome into an active complex. Activating signals include K^+^ efflux, Ca^2+^ influx, mitochondrial and lysosomal dysfunction, endoplasmic reticulum stress, and many others [9]. Inflammasome complex is assembled due to interaction of homotypic domains of NLRP3, ASC and procaspase-1, and as part of this complex, pro-caspase-1 auto-catalytically self-cleaves thus becoming active [10]. Then it cleaves the precursors of proinflammatory cytokines, pro-IL-1β and pro-IL-18, into their mature forms, which are released from the cells and trigger the cascade of inflammatory reactions. In addition, caspase-1 cleaves pore-forming protein gasdermin D (GSDMD) to N-and C-terminal fragments, where N-terminal fragment, due to its porin-like activity, inserts into the cell membrane, disrupts its integrity and induces inflammatory-related programmed cell death, called pyroptosis [11].

The role of the inflammasome in the pathogenesis of ALS has been demonstrated by studies on cell lines and transgenic mice models [12,13,14,15,16,17,18,19,20,21,22,23,24], whereas the number of similar studies in ALS patients is limited. Post-mortem studies of ALS patients have demonstrated elevated expression levels of NLRP3, ASC, caspase-1, IL-18 and GSDMD in tissues of CNS [13,25], and indicated pyroptosis as a potential mechanism of neuronal death [25]. Inflammasome complex components and IL-1β and IL-18 were also detected in vivo in biological fluids of ALS patients: elevated levels of IL-18 [26], caspase-1 [27], and IL-1β [28,29] were found in ALS serum, and of IL-1β [27] and IL-18 [30] in ALS cerebrospinal fluid. Though performed on small cohorts, these studies highlight the possible role of inflammasome in neuroinflammation and progression of ALS. However, the inconsistency of the data obtained by different scientific groups determines the necessity of more studies.

An important part of the research in this field is identification of in vivo biomarkers in order to evaluate neuroinflammation/inflammasome activation and to enable earlier and more accurate diagnosis and stratification of ALS patients into groups. Such biomarkers could also help to predict disease progression, functional outcome and the efficacy of treatment options [31,32]. Patients’ blood is a source of peripheral blood mononuclear cells (PBMCs) which may be isolated by conventional methods. Importantly, they include populations of immune cells and, thus, the inflammasome activation pathway can be traced in these cells on different levels, including mRNA and protein expression of inflammasome components and inflammation-related proteins and subsequent secretion pathways of cytokines triggered by inflammasome activation. Furthermore, close relation of neuroinflammation and peripheral inflammation is demonstrated [33,34,35,36,37,38], and thus PBMCs may mirror proinflammatory expression patterns characteristic of CNS resident immune cells, and at the same time, unlike CNS cells, are easily accessible for in vivo sampling. Finally, PBMCs have been demonstrated to reflect pathological changes in the CNS cells, characteristic of neurodegenerative diseases, i.e., the accumulation of TDP43 protein in the cytoplasm of ALS motor neurons has also been detected in circulating lymphocytes and monocytes [39,40].

It should be noted that interpretation of PBMC studies may be complicated due to technical variations in methodological procedures applied to PBMCs. A number of studies report that expression of PBMC genes is heavily influenced by isolation protocols, storage conditions and culturing [41,42,43]. First, PBMC studies usually report the use of either non-cultivated or cultivated cells [44,45,46,47,48,49,50,51,52,53,54,55]. However, to exclude the effect of cultivation, non-cultivated and cultivated PBMCs should be processed in parallel, which evidently is not possible in many human studies due to the limited cell amount and other technical limitations of the experiments. Second, in studies of inflammation and inflammasome the question arises whether in the ex vivo cellular context, additional exogenous stimuli are necessary to initiate the expression of NLRP3 inflammasome sensor and the IL-1β and IL-18 precursors and to detect inflammasome activation. Of note, in most inflammasome studies involving immune cell lines and primary immune cell cultures, for this purpose bacterial polysaccharide (LPS) or, in neurodegenerative disease studies, aggregation-prone proteins/peptides are used as priming signals [44,45,46,47,48,49]. However, exposing the cells to these “artificial” agents may misrepresent experimental results and their application to the actual disease process.

Due to ambiguity in the literature and a lack of the studies investigating the NLRP3 inflammasome in parallelly processed samples of cultivated and non-cultivated PBMCs from patients with ALS and other neurodegenerative diseases, we aimed to investigate the potential impact of PBMC cultivation and LPS stimulation on the first essential step of NLRP3 inflammasome activation. Specifically, we have evaluated mRNA expression and protein accumulation of NLRP3 inflammasome components and inflammation-related proteins in PBMCs of ALS patients and their matched Healthy controls. In our pilot study we found that mRNA expression levels of most NLRP3 inflammasome components and related cytokines were elevated in non-cultivated PBMCs. However, this was not accompanied by a significant increase in protein expression. At the same time, we detected the differences in the secretion profiles of inflammasome-activated cytokines in patient plasma and cultivated PBMC supernatants. These data on PBMC handling conditions can be used in future studies evaluating PBMC-derived biomarkers of ALS neuroinflammation and inflammasome activation in larger patient cohorts.

## 2. Materials and Methods

### 2.1. Patients

Twenty-three patients with amyotrophic lateral sclerosis (ALS) were enrolled in the study at the Russian Center of Neurology and Neurosciences (Moscow, Russia). All cases were classified as sporadic ALS, with no reported family history of motor neuron disease. The diagnosis was established by a board-certified neurologist based on a comprehensive clinical and neurophysiological evaluation, in accordance with the Gold Coast criteria [56]. Disease severity and functional status were additionally assessed using the revised ALS Functional Rating Scale (ALSFRS-R). All patients received riluzole at the standard dose of 50 mg twice daily as disease-modifying therapy. The Healthy controls (HC) group comprised twenty volunteers matched to the ALS group by age and sex.

Exclusion criteria, applied to both the ALS and HC groups, were as follows: severe cardiovascular disease; pulmonary pathology; hepatic dysfunction; renal failure; autoimmune diseases in active or chronic phase (with the exception of asymptomatic autoimmune thyroiditis); autoinflammatory disorders; systemic lupus erythematosus; rheumatoid arthritis; connective tissue diseases; hematopoietic disorders (e.g., myelodysplastic syndrome); malignancies (or absence of remission within the preceding 5 years); recurrent or chronic viral infections (HIV, hepatitis B and C); recent acute infections; neurodegenerative diseases other than ALS; severe psychiatric disorders, cognitive impairment, or dementia; clinically significant abnormalities in routine clinical and biochemical blood tests; vaccination within the previous 3 months; stem cell therapy; surgical interventions within the previous month; use of immunostimulating or immunosuppressive medications within the previous 3 months (including corticosteroids, antineoplastic agents, antibiotics, IL-1β antagonists, and TNF-α inhibitors); pregnancy or breastfeeding; and current tobacco smoking. The study was conducted in accordance with the principles of the Declaration of Helsinki and was approved by the Ethics Committee of the Russian Center of Neurology and Neurosciences (Protocol No. 2-5/23, approved on 15 February 2023). All participants provided written informed consent prior to enrollment.

The demographic and clinical characteristics of ALS patients and Healthy controls are summarized in Table 1. All experiments were performed in balanced batches processed in parallel, each run including ALS patients and Healthy controls (typically a 5:5 ratio), so that disease status was not confounded with processing batch, reagent lot, day of isolation, or operator, and the ALS-vs.-HC comparisons are not expected to be influenced by batch effects. As this study was considered as pilot, blinding and randomizing were not applied.

### 2.2. Peripheral Blood Mononuclear Cells (PBMCs) Isolation and Cultivation

Venous blood of ALS patients and Healthy controls was collected in 9 mL tubes containing 3.8% sodium citrate (Vacuette, Greiner Bio-One, Kremsmünster, Austria), kept at room temperature and transported to the laboratory for processing within 2 h. PBMCs were extracted by centrifugation over a Ficoll-Paque density gradient (Ficoll-Paque Plus, GE Healthcare, Chicago, IL, USA), washed with phosphate buffer saline (PBS) three times, and counted with a hemacytometer. After that, PBMCs from each subject were divided into two batches, non-cultivated (Non-Cult) and cultivated (Cult). Non-Cult PBMCs were further processed for RNA isolation and Western blot. Cult PBMCs were resuspended in complete RPMI medium (2 mM L-glutamine, 2 mM penicillin-streptomycin, 10% Fetal bovine serum (FBS, Cytiva HyClone, South American Origin, Marlborough, MA, USA)), immediately plated in 6-well plates (Corning, Steuben County, New York, NY, USA) in a concentration 1 mln/mL and incubated at 37 °C in 5% CO_2_ cell culture incubator. After ~21–22 h, PBMCs were left unstimulated, or stimulated with 1 µg/mL lipopolysaccharide from Escherichia coli O26:B6 (LPS, Sigma Aldrich Fine Chemicals Biosciences, St. Louis, MA, USA) for 3 h. At the end of incubation, the PBMCs were centrifuged and culture supernatants were collected in Eppendorf tubes and stored at −80 °C until downstream assays. Cell viability in each sample was assessed by Trypan blue die exclusion, Cult PBMCs were counted with a hemacytometer, and further processed for RNA isolation and Western blot. The same batch of serum and medium were used in all experiments.

### 2.3. Reverse Transcription and Real-Time Quantitative Polymerase Chain Reaction (RT-qPCR)

Total RNA was extracted from 3 mln PBMCs (Non-Cult or Cult) using the column-based total RNA isolation kit (RNA solo, Evrogen, Moscow, Russia) supplemented with a DNase step. RNA concentration was measured and RNA quality (260 nm/280 nm, 260 nm/230 nm ratios) was confirmed on NanoDrop ND-1000 (Thermo Scientific, Waltham, MA, USA). A total of 500 ng of isolated RNA was reverse transcribed in the presence of 20 µM oligo-dT using Mint Reverse Transcriptase (Evrogen, Moscow, Russia) following the manufacturer’s instructions. In total, 10 ng of cDNA was amplified using qPCRmix-HS SYBR (Evrogen, Moscow, Russia) in the Light Cycler 96 (Roche, Basel, Switzerland). Gene-specific primers were designed using the NCBI gene (https://www.ncbi.nlm.nih.gov/gene/, accessed on 10 July 2025) and Integrated DNA technologies (https://www.idtdna.com/pages/tools/oligoanalyzer, accessed on 10 July 2025) websites; their sequences are given in Supplementary Table S1. qPCR amplification efficiency was tested by performing 2-fold or 5-fold serial dilutions of cDNA samples across 5 logs in triplicates, and was approaching 2.0 for each primer tested. The amplification program was performed using the following cycling conditions: initial denaturation for 5 min at 95 °C, followed by 45 cycles of 10 s denaturation at 95 °C, 20 s annealing at 59 °C and 25 s of elongation at 72 °C. The presence of a single amplified product was confirmed by DNA melting curve analysis, which showed a single melting curve peak. Assays were run in triplicate. mRNA expression was normalized to the mRNA levels of ribosomal protein L13a, RPL13A (NM_012423.2) used as housekeeping gene. Fold changes in the expression of genes of interest were calculated using ΔΔCt method.

### 2.4. Western Blot

Non-Cult and Cult PBMCs (1 mln cells for each sample) were washed with PBS buffer and lysed with Laemmli buffer (50 mM Tris-HCl pH 6.8, 2% SDS, 10% glycerol, 2% β-mercaptoethanol, 0.025% bromophenol blue) with an addition of protease inhibitor cocktail (cOmplete Mini, EDTA-free, Sigma-Aldrich, St. Louis, MA, USA) at 95 °C for 10 min. The samples were analyzed by electrophoresis in 8, 12, or 15% SDS-PAGE under reducing conditions, and the proteins from the gel were transferred to PVDF (Thermo Scientific, Waltham, MA, USA) or nitrocellulose (Amersham-GE Healthcare, Chicago, IL, USA) membranes using X cell II blot module for wet transfer (Invitrogen, Carlsbad, CA, USA). Membranes were blocked in PBS-T buffer (PBS, 0.05% Tween 20, 5% skim milk) for 2 h followed by overnight incubation with antibodies specific for: NLRP3 (AG-20B-0014-C100), ASC (AG-25B-0006-C100), caspase-1 (AG-20B-0048-C100) (all from Adipogen, San Diego, CA, USA), IL-1β (ab216995, Abcam, Cambridge, UK), IL-18 (67775T, Cell Signaling, Danvers, MA, USA) diluted in blocking buffer containing PBS with 0.05% Tween-20 and 5% skim milk or BSA in concentrations recommended by manufacturers. The antibodies have been validated in the cell line negative for expression of inflammasome complex proteins and IL-1β (Supplementary Figure S1). Based on this validation, the expected band sizes for the analyzed proteins were predicted, and non-specific bands were excluded from the analysis of Western blots of PBMC samples.

Horseradish peroxidase-conjugated (HRP-conjugated) goat antibodies to rabbit and mouse immunoglobulins (Jackson, ImmunoResearch, West Grove, PA, USA) were used as secondary antibodies. Membrane complexes were detected using the chemiluminescent reagent Clarity™ Western ECL Substrate (BioRad, Hercules, CA, USA) and the signal was recorded on a ChemiDoc instrument (BioRad, Hercules, CA, USA). The data were processed using ImageJ 154 (https://imagej.net/ij/, accessed on 2 April 2026). After inflammasome proteins and IL-1β-specific staining, blots were stripped and restained with monoclonal anti-β-actin murine antibodies (clone AC-74, Sigma-Aldrich Fine, St. Louis, MA, USA) for 1 h and then with secondary HRP-conjugated goat antibodies to mouse immunoglobulins (Jackson, ImmunoResearch, West Grove, PA, USA). For relative quantification, densitometry analysis of signals was performed by ImageJ software and normalized to corresponding β-actin signal.

### 2.5. ELISA

Venous blood of ALS patients and Healthy controls was collected in 9 mL tubes containing 3.8% sodium citrate (Vacuette, Greiner Bio-One, Kremsmünster, Austria), kept at room temperature and transported to the laboratory for processing within 2 h. Plasma samples were isolated by centrifugation at 300× *g* for 10 min, aliquoted in Eppendorf tubes and stored at −80 °C till further analysis.

IL-1β and IL-18 concentration in plasma samples and culture supernatants from PBMCs was determined by sandwich immunoassays, Interleukin-1 beta-EIA-BEST and Interleukin-18-EIA-BEST, correspondingly, according to the manufacturer’s instructions (both Vector-Best, Novosibirsk, Russia). The samples were analyzed in two technical repeats. To quantify the cytokine concentrations, the absorbance of samples was measured at 450 and 620 nm using a plate reader (Tecan Trading, Mannedorf, Switzerland), and the 620 nm absorbance value (background signal from the instrument) was subtracted from the 450 nm absorbance value.

### 2.6. Statistics and Software

Quantitative data were tested for normality by Shapiro–Wilk test. Several variables were non-normally distributed and group sizes were unequal, so non-parametric methods were used throughout. Data were reported as median and interquartile range [Q1, Q3], with exact number of biological replicates (n; one sample per ALS patient or Healthy control) given in Supplementary Tables S2–S4. Omnibus differences among the multiple PBMC conditions were first assessed with the Kruskal–Wallis test. For pairwise comparisons, independent groups (ALS vs. Healthy controls) were compared with the two-sided Mann–Whitney U test (exact test was used where computationally feasible; otherwise, the normal approximation with continuity correction was applied), and within-subject comparisons of LPS-stimulated vs. unstimulated cultivated PBMCs, for which matched samples were available, were performed with the two-sided Wilcoxon signed-rank test. Comparisons between non-cultivated and cultivated PBMCs, although derived from the same donors, were analyzed as independent samples because not all donors contributed to every condition; given the large magnitude of the cultivation effect, it did not affect the conclusions. Categorical variables were compared with Fisher’s exact test. To account for multiple testing, *p*-values were adjusted within each family of comparisons (the panel of genes or proteins examined for a given contrast) with the Benjamini–Hochberg false-discovery-rate procedure (reported as q). Effect sizes were reported as the rank-biserial correlation, and 95% confidence intervals for the difference in medians were estimated with the Hodges–Lehmann method (Supplementary Tables S2–S4). An adjusted q < 0.05 was considered statistically significant. Analyses were performed using GraphPad Prism 8.4.3 (GraphPad Software Inc., San Diego, CA, USA) and Python 3.11 for multiplicity correction and effect-size estimation.

## 3. Results

### 3.1. Literature Overview of Human PBMC-Based Studies of NLRP3 Inflammasome in Neurodegenerative Diseases

First, we tried to determine an optimal protocol for handling PBMCs based on the known studies of biomarkers of NLRP3 inflammasome expression and activation in PBMCs from patients with neurodegenerative diseases. Only one relevant ALS study was found, so we also added studies on Alzheimer’s disease (AD) and Parkinson’s disease (PD) to the analysis, which was motivated by the evidence of the NLRP3 inflammasome role in their pathogenesis and by some similarities of putative mechanisms of inflammasome activation in ALS and these disorders [57,58,59,60]. Table 2 summarizes our findings containing the data of nine PD trials, three AD trials and one ALS trial. Most of the studies were performed in PBMCs, and three of them in monocytes isolated from PBMCs either upon their short-term cultivation by washing out non-adherent cells (#1, 7), or by negative selection with the use of specialized kit (#13). We were focused on the following data: mRNA expression of NLRP3 inflammasome components, IL-1 β and IL-18 genes, which was evaluated by RT-qPCR and RNA sequencing; protein expression of NLRP3 inflammasome components, IL-1β and IL-18, which was conventionally evaluated by Western blot and in individual studies, by confocal microscopy/immunochemistry (proteins co-localization) and flow cytometry (proteins co-expression); secretion of IL-1β and IL-18 to plasma/serum of study participants or to cultivated PBMC supernatants, which was evaluated by ELISA including high-sensitive and multiplexed high-throughput assays (Table 2). Two studies used the specific method, AMNIS FlowSight, for evaluation of ASC speck complexes’ formation, which is an indicator of the assembly of the active inflammasome complex [61] (#3, 10, Table 2).

The following observations were made from the analysis of the studies:

(1) Among thirteen studies, nine were performed in cultivated PBMCs, and only three were performed in non-cultivated PBMCs (#2, 9, 11), and one in non-cultivated monocytes (#13). No study has compared non-cultivated PBMCs with cultivated cells.
(2) In AD trials, the study on non-cultivated PBMCs (#2) claimed the higher levels of inflammasome components expression and secretion of mature caspase-1, IL-1β and GSDMD in patients in comparison to controls, whereas the same difference was observed in cultivated PBMCs only in conditions of stimulation with LPS with subsequent stimulation with amyloid-β peptide; AD and HC PBMCs, both unstimulated and stimulated with sole LPS or amyloid-β peptide, demonstrated similar patterns of inflammasome expression/activation (studies #1, 3).
(3) The conclusions from PD trials were more controversial, likely due to the larger number of the trials analyzed. In most studies, stimulation of cultivated PBMCs with LPS or different forms of α-synuclein (monomers, oligomers, or fibrils) led to the higher expression/activation of the NLRP3 inflammasome in both PD patients and HC groups (#4–8, #10–12). However, LPS/α-synuclein stimulation did not always induce the expected elevated production of the inflammasome complex components and increased inflammasome activation. In study #10, LPS stimulation did not induce ASC speck formation, as expected. In study #12, stimulation of PD PBMCs with α-synuclein monomers or oligomers did not increase the levels of IL-1β and IL-18 in PBMCs supernatants, as it did for HC PBMCs. Regarding differences in inflammasome activation between PD patients and HC groups, the results of different studies were controversial. In some trials no differences were found in any of the tested conditions (unstimulated or stimulated PBMCs) (#8, 10). In other trials, PD PBMCs showed higher NLRP3 expression/activation in both unstimulated and stimulated conditions (#6). Other studies found that unstimulated PD PBMCs had higher NLRP3 expression/activation than unstimulated HC PBMCs; however, treatment of HC PBMCs with LPS or α-synuclein eliminated this difference (#12). Study #4 found no difference between groups in unstimulated PBMCs, but a difference appeared with stimuli treatment. Trial #5 demonstrated that IL-1β level in supernatants of PD patients’ PBMCs was even lower as compared to HC PBMC supernatants. Trials with non-cultivated PBMCs (#9, 11) generally showed that PD PBMCs demonstrated higher inflammasome activation than HC PBMCs. However, in study #11 the levels of IL-1β and IL-18 were equal in the plasma of PD patients and HCs.
(4) In the ALS trial, which was performed on non-cultivated monocytes, ALS monocytes showed higher mRNA expression levels of NLRP3 and IL-1β, but IL-1β was not detected in either ALS or HC serum.

After analyzing the data, we formulated three principal questions for further evaluation of inflammasome expression in ALS patients PBMCs, which are currently unclear:

- Which conditions of PBMC handling are optimal and how does cultivation influence expression of NLRP3 inflammasome and inflammasome-related cytokines?
- Is LPS (or other exogenous stimulus) absolutely necessary to detect NLRP3 inflammasome components and inflammation-related cytokines expression, or can the study be performed in unstimulated PBMCs, which, in theory, should more reliably reflect the pathological processes occurring in the patient’s body and not the effect of exogenous stimulus?
- Can handling conditions of PBMCs influence the detection of differences between HC and ALS patients?

Looking for the answers, we initiated a pilot trial of NLRP3 inflammasome and inflammation-related protein expression focused on testing the handling conditions of PBMCs from ALS patients and Healthy controls.

### 3.2. Gene Expression of NLRP3 Inflammasome Components and Inflammation-Related Proteins in PBMCs: Effects of Cell Cultivation, Lipopolysaccharide Stimulation and ALS Disease

As mentioned above, the activation of NLRP3 complex is a two-step process. The first step involves the induction of gene expression of complex components and effector proteins (IL-1β and IL-18 cytokines and GSDMD) in response to a priming signal. In our study, we analyzed the expression levels of these genes. The expression of *NLRP3* and *pro-IL1-β* genes depends most on the priming signal, which activates NF-κB signaling [10]. Meanwhile *ASC*, *pro-caspase-1*, *pro-IL-18* and *GSDMD* genes can be constitutively expressed in immune cells (steady-state expression) [63,64,65,66], though to varying degrees their expression levels can be upregulated in response to priming signals. In addition to evaluating the mRNA expression of the genes directly related to inflammasome activation, we also examined the mRNA expression of *IL-18BP* and *IL-6*. IL-18BP (IL-18 Binding Protein) is an IL-18 antagonist that binds IL-18 with high affinity, thereby inhibiting its activity [67]. It is also constitutively expressed in the immune cells [68,69]. We included *IL-6*, the expression of which depends on NF-κB signaling [70], in the analysis because it is a common target of IL-1 and IL-18 downstream signaling [71] and a hallmark of neurodegenerative disorders [72].

**Figure 1:**
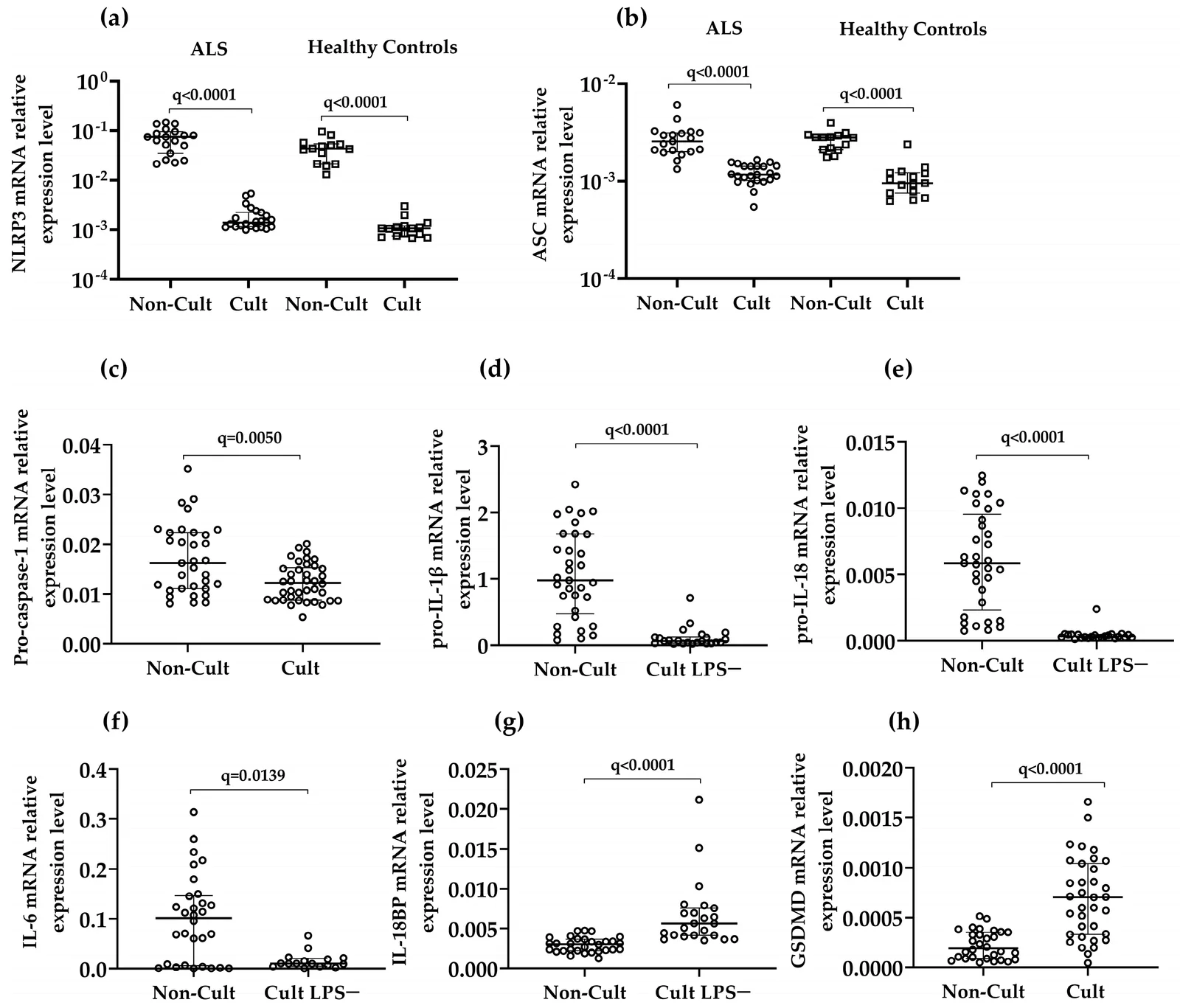
Comparison of mRNA expression levels of genes encoding NLRP3 inflammasome components and inflammation-related proteins in non-cultivated vs. cultivated PBMCs of ALS patients and Healthy controls. (**a**) NLRP3 expression of Non-Cult ALS PBMCs vs. Cult (LPS− and LPS+) ALS PBMCs, and of Non-Cult HC PBMCs vs. Cult (LPS− and LPS+) HC PBMCs; (**b**) ASC expression of Non-Cult ALS PBMCs vs. Cult (LPS− and LPS+) ALS PBMCs, and of Non-Cult HC PBMCs vs. Cult (LPS− and LPS+) HC PBMCs; (**c**) pro-caspase-1 expression of Non-Cult (ALS and HC) PBMCs vs. Cult (LPS− and LPS+) (ALS and HC) PBMCs; (**d**) pro-IL-1β expression of Non-Cult (ALS and HC) PBMCs vs. Cult (LPS−) (ALS and HC) PBMCs; (**e**) pro-IL-18 expression of Non-Cult (ALS and HC) PBMCs vs. Cult (LPS−) (ALS and HC) PBMCs; (**f**) IL-6 expression of Non-Cult (ALS and HC) PBMCs vs. Cult (LPS−) (ALS and HC) PBMCs; (**g**) IL-18BP expression of Non-Cult (ALS and HC) PBMCs vs. Cult (LPS−) (ALS and HC) PBMCs; (**h**) GSDMD expression of Non-Cult (ALS and HC) PBMCs vs. Cult (LPS− and LPS+) (ALS and HC) PBMCs. One point represents relative expression level of a gene of interest normalized to *RPL13A* expression in individual subject, the horizontal line indicates the median, the lines stretching from the median indicate interquartile range. q values are shown on the graph, q < 0.05 were considered significant, Mann–Whitney U-tests with Benjamini–Hochberg false-discovery-rate procedure applied for multiple testing. Per group n values, effect sizes and 95% CI are reported in Supplementary Table S2. Non-Cult—non-cultivated PBMCs, Cult—cultivated PBMCs, LPS−—LPS-untreated PBMCs, LPS+—PBMCs treated with 1 µg/mL LPS for 3 h, ALS—amyotrophic lateral sclerosis, HC—Healthy controls.

**Figure 2:**
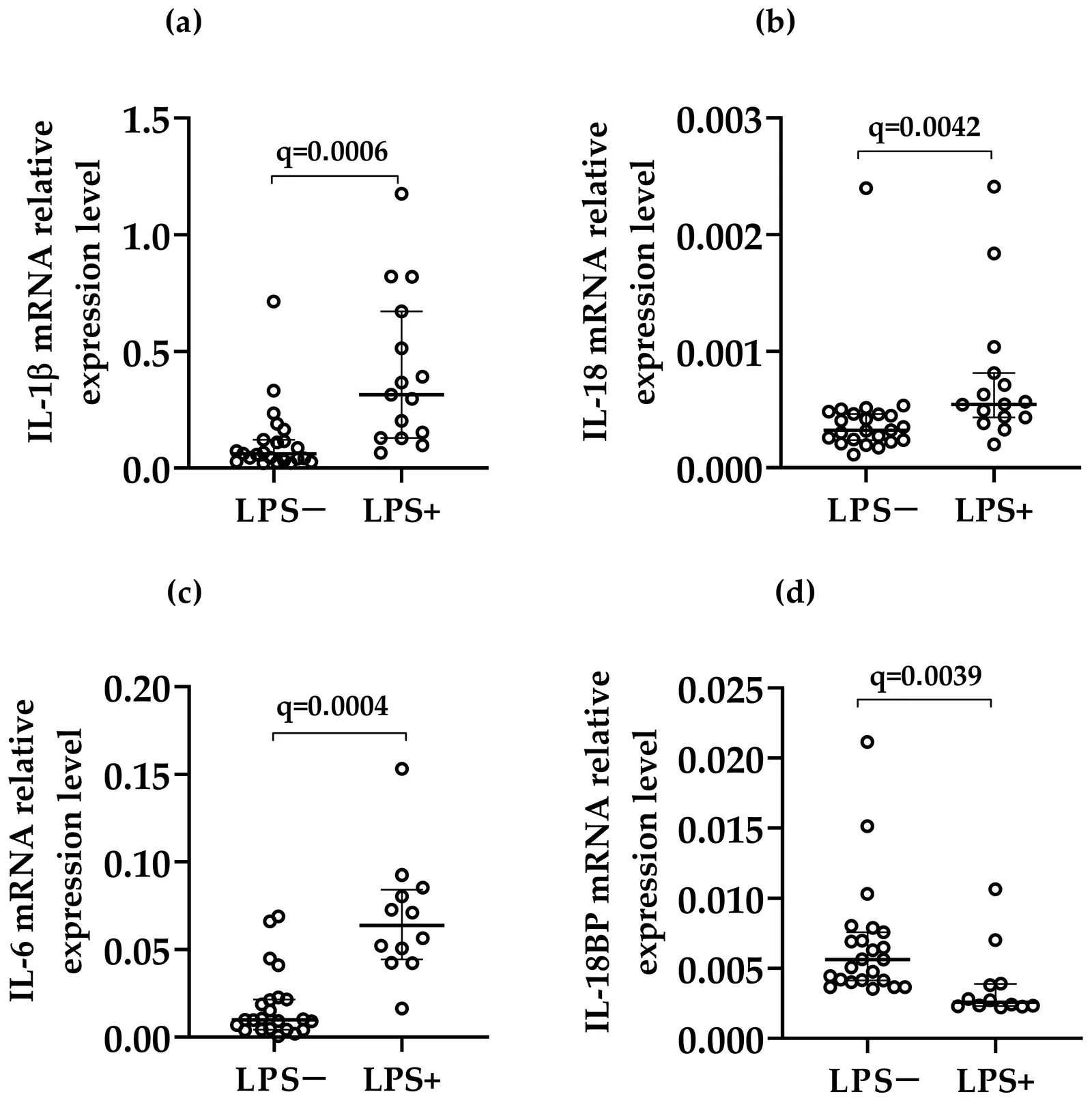
LPS treatment changes mRNA expression levels of inflammation-related proteins in cultivated PBMCs of ALS patients and Healthy controls. Comparison was performed in the groups of Cult LPS− PBMCs (ALS and HC) vs. Cult LPS+ PBMCs (ALS and HC) for mRNA expression levels of: (**a**) IL-1β, (**b**) IL-18, (**c**) IL-6, (**d**) IL-18BP. One point represents relative expression level of a gene of interest normalized to *RPL13A* expression in individual subject, the horizontal line indicates the median, the lines stretching from the median indicate interquartile range. q values are shown on the graphs, q < 0.05 were considered significant, Mann–Whitney U-tests with Benjamini–Hochberg false-discovery-rate procedure applied for multiple testing. Per group n values, effect sizes and 95% CI are reported in Supplementary Table S2. Cult—cultivated PBMCs, LPS−—LPS-untreated PBMCs, LPS+—PBMCs treated with 1 µg/mL LPS for 3 h, ALS—amyotrophic lateral sclerosis, HC—Healthy controls.

**Figure 3:**
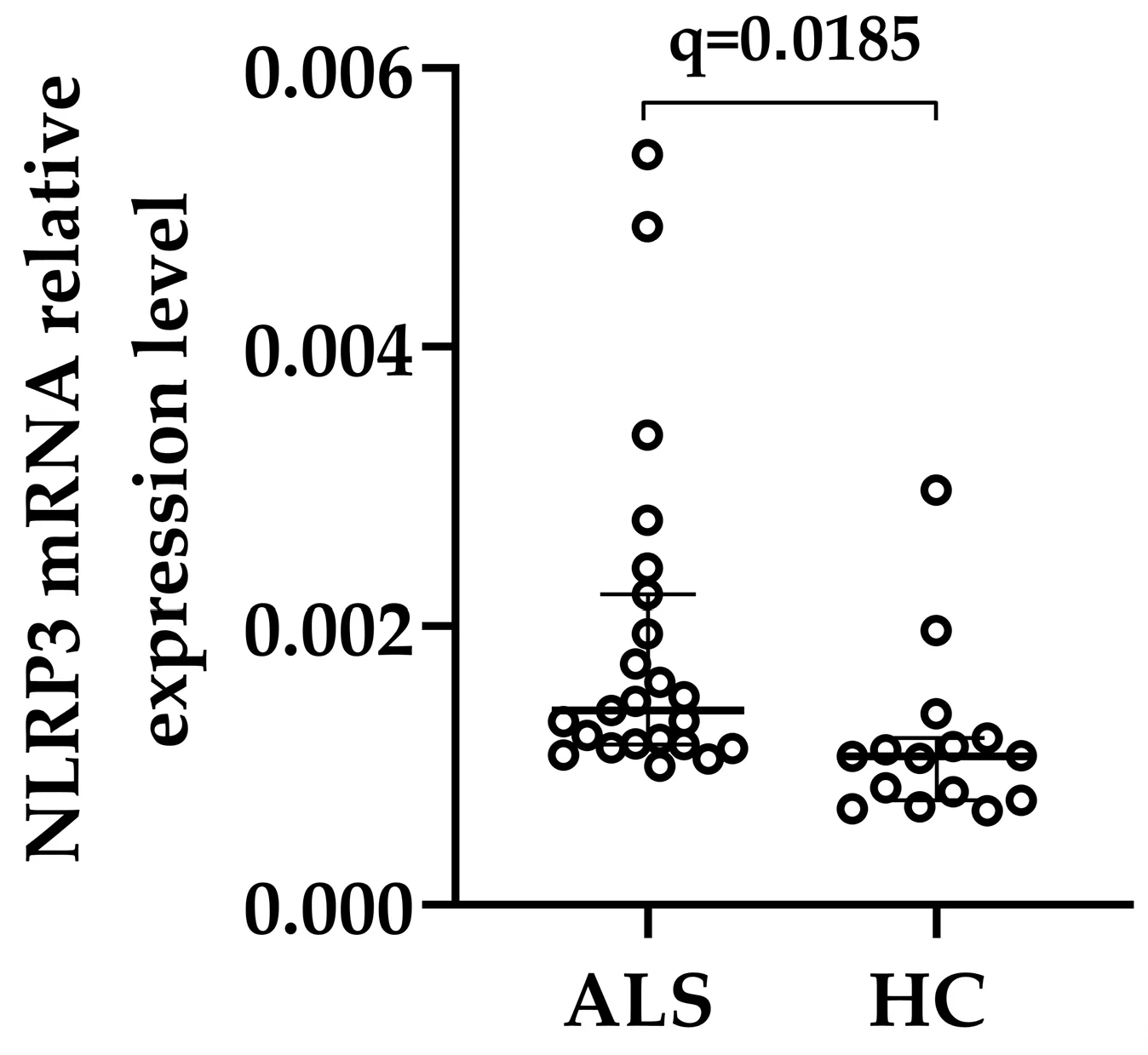
Cultivated PBMCs of ALS patients have elevated levels of NLRP3 mRNA expression. mRNA expression levels of NLRP3 in Cult (LPS− and LPS+) ALS PBMCs vs. Cult (LPS− and LPS+) HC PBMCs. One point represents relative expression level of a gene of interest normalized to *RPL13A* expression in individual subject, the horizontal line indicates the median, the lines stretching from the median indicate interquartile range. q values are shown on the graph, q < 0.05 were considered significant, Mann–Whitney U-tests with Benjamini–Hochberg false-discovery-rate procedure applied for multiple testing. Per group n values, effect sizes and 95% CI are reported in Supplementary Table S2. Cult—cultivated PBMCs, LPS−—LPS-untreated PBMCs, LPS+—PBMCs treated with 1 µg/mL LPS for 3 h, ALS—amyotrophic lateral sclerosis, HC—Healthy controls.

To analyze the mRNA expression levels of the chosen genes, PBMCs were isolated from the whole blood of ALS patients and Healthy controls (HC) with Ficoll-Paque using density gradient centrifugation. PBMCs were divided into two batches: non-cultivated (Non-Cult) and cultivated (Cult). RNA isolation was immediately performed on the Non-Cult PBMCs, while Cult PBMCs were subjected to 21–22 h cultivation. Subsequently, Cult PBMCs were treated with LPS for 3 h, or left untreated. After stimulation was complete, RNA was isolated from Cult PBMCs. Furthermore, the mRNA expression levels of the NLRP3 inflammasome complex genes (*NLRP3*, *ASC*, *pro-caspase-1*) and the inflammation-related protein genes (*IL-1β*, *IL-18*, *IL-18BP*, *GSDMD*, *IL-6*) were analyzed by Real-time qPCR in both PBMC batches. The obtained data are summarized in the Supplementary Table S2 (adjusted *p*-values are reported as q and those illustrating statistically significant differences are shown in red), and Figure 1, Figure 2 and Figure 3 illustrate significant effects found.

Figure 1 illustrates the difference between Non-Cult and Cult PBMC batches which have been compared in different combinations: Non-Cult vs. Cult LPS−, Non-Cult vs. Cult LPS+, or Non-Cult vs. Cult (LPS− and LPS+) (Supplementary Table S2). With the exception of Figure 1a,b (NLRP3 and ASC expression), ALS and HC PBMCs were merged in one group as no statistically significant differences were found for ALS vs. HC). At least for one combination of groups, mRNA expression levels of NLRP3, ASC, caspase-1, IL-1β, IL-18 and IL-6 were found statistically significant higher in Non-Cult PBMCs. The most dramatic difference was detected for NLRP3 expression, where the difference reached 40–50 times (Figure 1a). Expression levels of IL-1β, IL-18 and IL-6 were approximately an order of magnitude higher in Non-Cult PBMCs than in Cult PBMC (Figure 1d–f; the graphs illustrate the data for Non-Cult vs. Cult LPS− groups, without inclusion of data for LPS-treated PBMCs, as LPS treatment significantly upregulated their expression levels (Supplementary Table S2 and Figure 2). The least pronounced difference was detected for ASC expression level: in Cult PBMCs it was decreased only 2–3 fold (Figure 1b), and for pro-caspase-1 expression level, the downregulation effect was 25% (Figure 1c). Interestingly, in the case of IL-18BP and GSDMD, the opposite effect was observed, and cultivation of PBMCs upregulated their mRNA expression levels 2–4 fold (Figure 1g,h).

Next, we evaluated the effect of bacterial lipopolysaccharide (LPS) in Cult PBMCs. LPS is expected to act as a priming signal in two-step NLRP3 inflammasome activation and is able to upregulate expression of genes of inflammasome components and cytokines [73]. Some studies show that expression of inflammasome components may be elevated in immune cell lines in the absence of LPS or any other priming stimuli, with subsequentNLPR3 inflammasome activation under these conditions [74]. We have observed the expected effect of LPS treatment on cytokine gene expression levels (Supplementary Table S2): 5-fold increase was shown for IL-1β (Figure 2a), 1,7-fold increase, for IL-18 (Figure 2b), and 6-fold increase, for IL-6 (Figure 2c). Interestingly, LPS negatively affected IL-18BP gene expression level, reducing it 2-fold (Figure 2d, Supplementary Table S2). Although the stimulatory effect of LPS was expected based on literature data, it was not observed for NLRP3 expression levels, possibly due to the small sample size (Supplementary Table S2). The levels of pro-caspase-1 and GSDMD were unchanged in response to LPS (Supplementary Table S2). Of note, even in the absence of LPS, we have observed significant expression levels of all the components of NLRP3 inflammasome complex and inflammasome-activated cytokines (Supplementary Table S2).

Last but not least, we have analyzed whether ALS disease has an effect on gene expression levels of NLRP3 inflammasome and inflammation-related proteins. Among all the subgroups compared, the only statistically significant effect was observed for NLRP3 gene: in Cult ALS PBMCs—1,4-fold increase was detected in comparison to HC (Supplementary Table S2, Figure 3).

Thus, in our studies of mRNA expression of inflammasome- and inflammation-related proteins, the most pronounced effect of cell handling conditions was associated with PBMC cultivation. The second effect we observed was the expected elevation of expression levels of cytokines in response to LPS stimulation. The effect of ALS disease was seen only forNLRP3 mRNA expression levels and only in cultivated PBMCs.

### 3.3. Protein Expression of NLRP3 Inflammasome Components and IL-1β in PBMCs of ALS Patients and Healthy Controls

**Figure 4:**
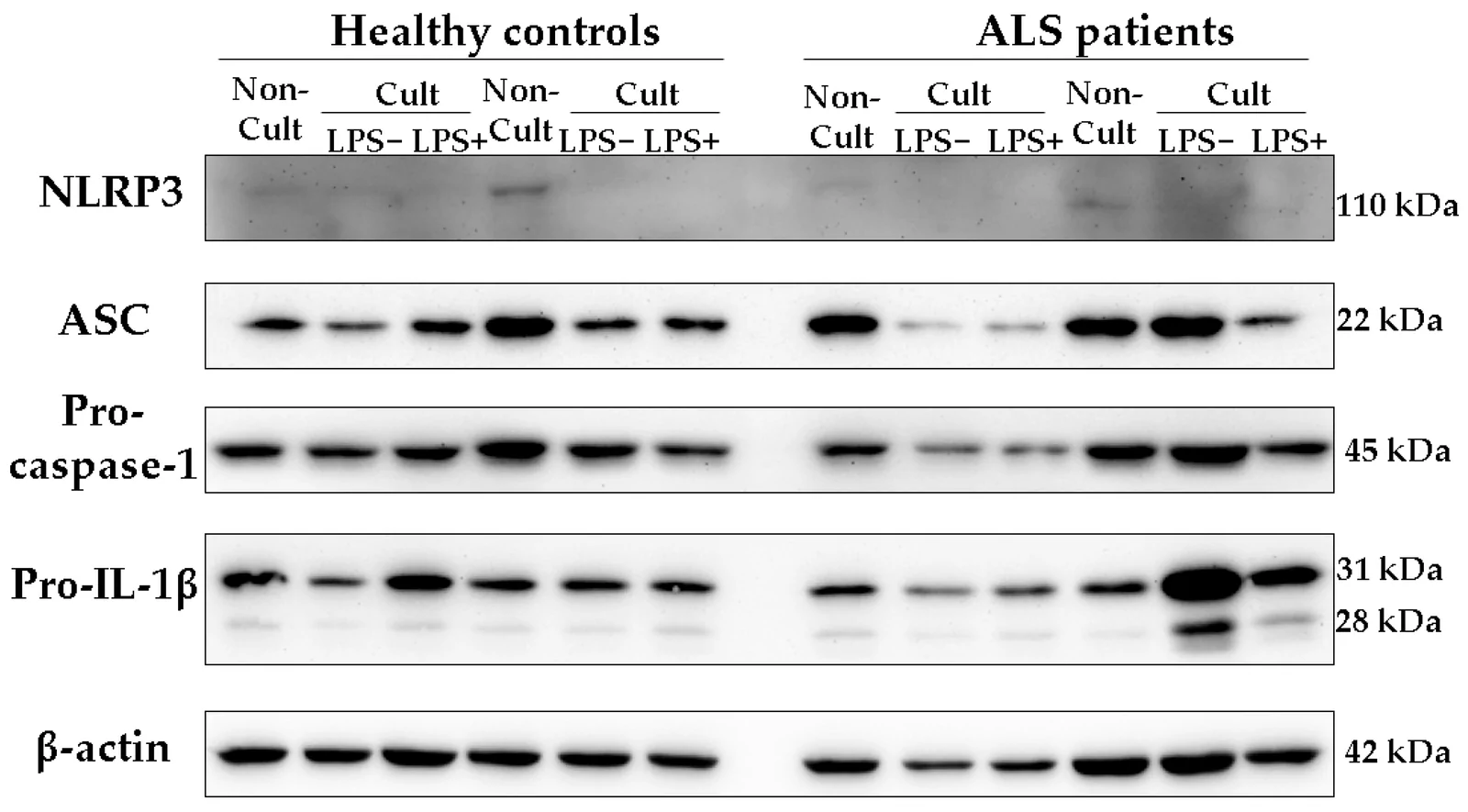
Western blot of NLRP3 inflammasome components and IL-1β in non-cultivated and cultivated PBMCs of ALS patients and Healthy controls. The protein levels of NLRP3, ASC, procaspase-1 and pro-IL-1β were analyzed by Western blot in PBMCs lysates from two representative Healthy controls and two representative ALS patients. Blots were stained with specific antibodies, and then stripped and re-stained with anti-β-actin antibodies; relevant molecular mass of analyzed proteins is given to the right (kDa). The data on relative expression levels of analyzed proteins and their statistical analysis are illustrated by Supplementary Figure S2. Antibody validation was performed in HEK293T cells and is reported on Supplementary Figure S1. Non-Cult—non-cultivated PBMCs, Cult—cultivated PBMCs, LPS−—LPS-untreated PBMCs, LPS+—PBMCs treated with 1 µg/mL LPS for 3 h, ALS—amyotrophic lateral sclerosis patients.

To ensure that the mRNAs of NLRP3 inflammasome genes are expressed into corresponding proteins and to find out if differences in mRNA expression levels observed between Non-Cult and Cult PBMCs will be reproduced on the protein level, we performed Western blot on PBMC lysates using antibodies specific for NLPR3, ASC, caspase-1, IL-1β and IL-18 (Figure 4, Western blot of two representative samples of ALS patients and HC). The data on relative protein expression levels were normalized to β-actin and are represented in Supplementary Table S3 and on Supplementary Figure S2. IL-18 protein expression was beyond the detection limit. This was also observed in the other ALS study [75], and could be probably associated with low antibody specificity. The only statistically significant difference was observed for pro-IL-1β protein expression levels between Non-Cult PBMC and Cult (LPS− and LPS+) PBMC of both ALS and HC groups: in Cult ALS PBMC expression levels increased 2-fold, and in Cult HC PBMCs, 3,5-fold (Supplementary Table S3, Supplementary Figure S2d). So, with exception of this effect, the difference observed between mRNA expression level of NLRP3 inflammasome components and IL-1β in Non-Cult and Cult PBMCs was not translated on the protein expression level. We also have not observed any stimulatory LPS effect on the accumulation of analyzed proteins, as well as any difference between ALS patients and Healthy controls.

### 3.4. IL-1β and IL-18 Secretion Levels in Plasma and Cultivated PBMC Supernatants of ALS Patients and Healthy Controls

**Figure 5:**
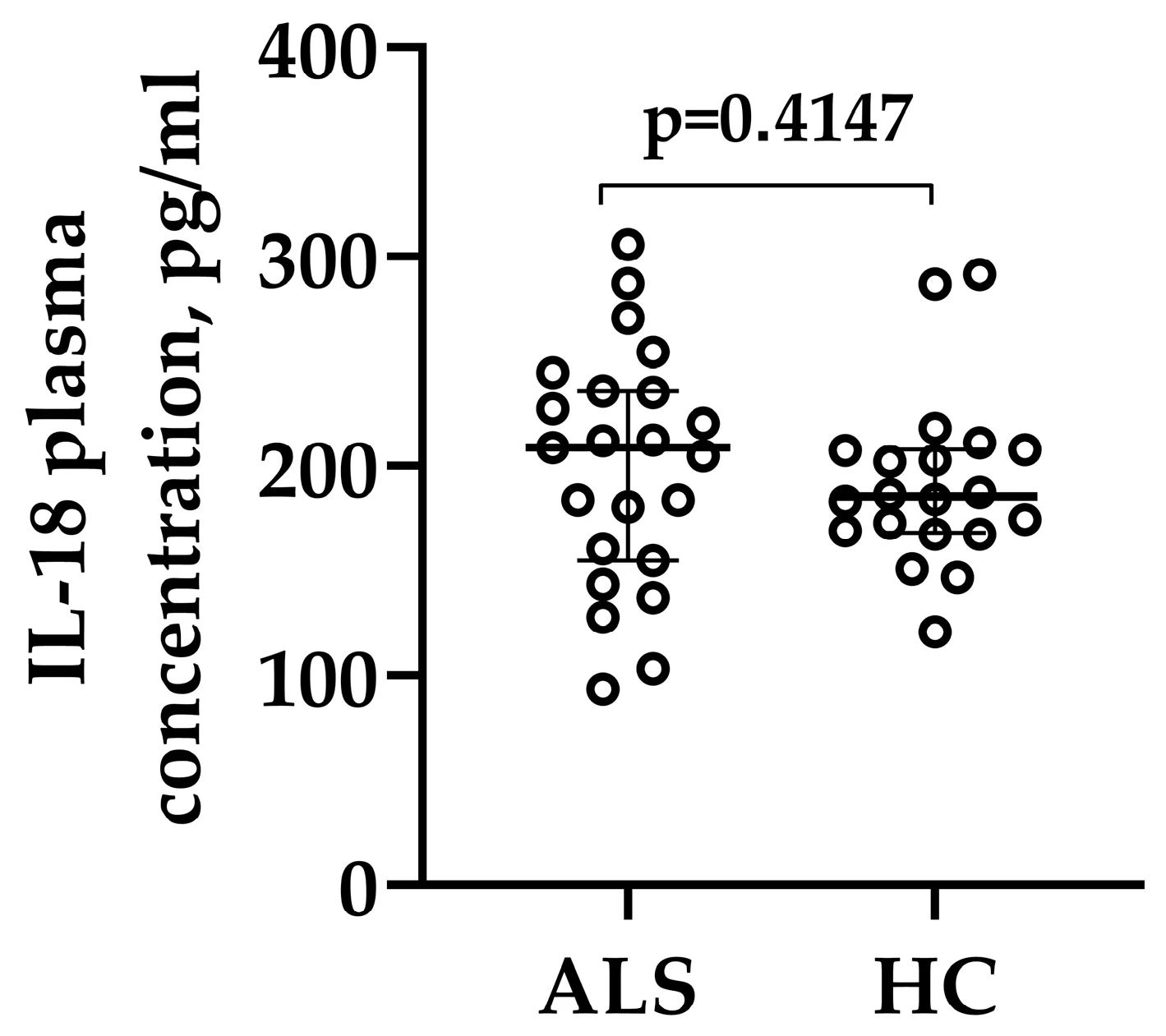
IL-18 plasma levels of ALS patients compared to HC. IL-18 concentration (pg/mL) was measured by ELISA in plasma of ALS patients and HC. One point represents IL-18 level in plasma of individual subject, the horizontal line indicates the median, the lines stretching from the median indicate interquartile range. *p*-value is shown on the graph Mann–Whitney U-test. Per group n values, effect sizes and 95% CI are reported in Supplementary Table S4. ALS—amyotrophic lateral sclerosis, HC—Healthy controls.

**Figure 6:**
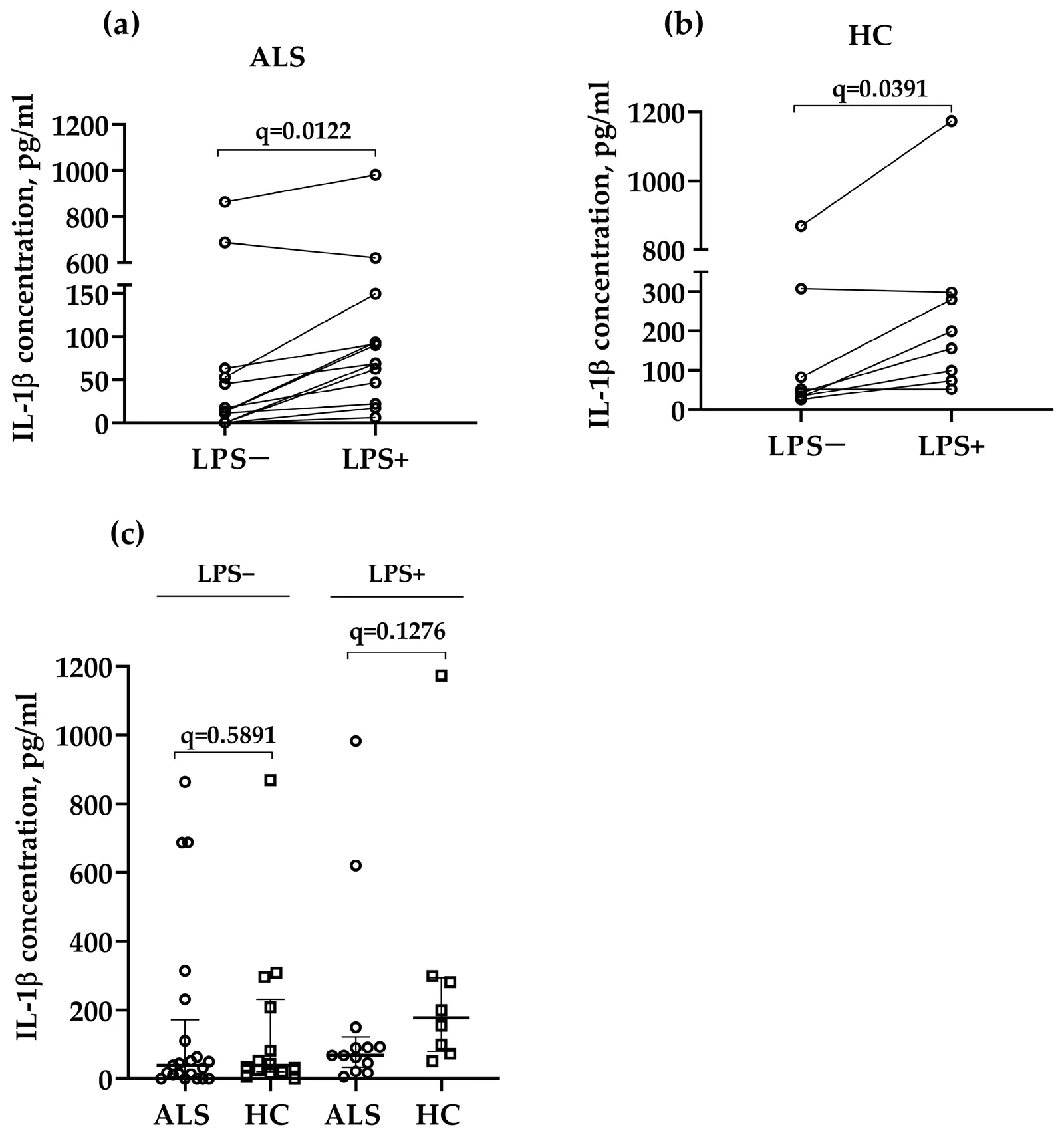
IL-1β levels in cultivated PBMC supernatants of ALS compared to HC. IL-1β concentration (pg/mL) was measured by ELISA in supernatants of cultivated PBMCs of ALS patients and HC. (**a**,**b**) Within-subject comparisons of IL-1β concentration in supernatants of cultivated mock-treated (LPS−) vs. LPS-treated (LPS+) PBMCs of (**a**) ALS and (**b**) HC. q-values are shown on the graph, q < 0.05 were considered significant, two-sided Wilcoxon signed-rank test with Benjamini–Hochberg false-discovery-rate procedure applied for multiple testing. (**c**) IL-1β supernatant concentrations of cultivated LPS− PBMCs of ALS compared to LPS− PBMCs of HC and of cultivated LPS+ PBMCs of ALS compared to LPS+ PBMC of HC. One point represents IL-1β level in supernatant of cultivated PBMCs isolated from individual subject, the horizontal line indicates the median, the lines stretching from the median indicate interquartile range. q-values are shown on the graph, q < 0.05 were considered significant, Mann–Whitney U-tests with Benjamini–Hochberg false-discovery-rate procedure applied for multiple testing. Per group n values, effect sizes and 95% CI are reported in Supplementary Table S4. LPS−—LPS-untreated PBMCs, LPS+—PBMCs treated with 1 µg/mL LPS for 3 h, ALS—amyotrophic lateral sclerosis, HC—Healthy controls.

Finally, the concentrations of soluble IL-1β and IL-18 cytokines, which production and secretion are associated with the activation of NLRP3 inflammasome, were analyzed in plasma of ALS patients and HC, as well as in ALS and HC PBMC culture supernatants by enzyme-linked immunosorbent assays (ELISA) (Supplementary Table S4 and Figure 5 and Figure 6). It should be noted that ELISA analysis does not provide clear differentiation between precursor and cleaved forms of these cytokines, and cannot be a direct measure of NLRP3 inflammasome activation, unless it is highly specific for the detection of the cleaved cytokine forms. However, the fluctuations of secreted cytokine levels may indirectly indicate the changes in proinflammatory status of the patients. In our study, we found that plasma IL-1β was below kit detection level in both ALS patients (n = 23) and Healthy controls (n = 20), while equal levels of IL-18 were detected in plasma of both groups (Figure 5).

The opposite phenomenon was observed in cultivated PBMC supernatants, where IL-18 was at background levels in all the subgroups analyzed, ALS LPS− (n = 21), ALS LPS+ (n = 13), HC LPS− (n = 14), HC LPS+ (n = 8). At the same time, secreted IL-1β was detected in supernatants of both ALS and HC PBMCs (Figure 6, Supplementary Table S4). Figure 6a,b illustrates the effect of LPS treatment analyzed in available matched samples of LPS− vs. LPS+ cultivated PBMCs by within-subject comparisons with the two-sided Wilcoxon signed-rank test. Thus, LPS was demonstrated to upregulate the level of secreted IL-1β, which was in accordance with the increased IL-1β mRNA expression levels observed in LPS-treated PBMCs (Supplementary Table S2, Figure 2a). Yet, comparison of IL-1β concentration levels between ALS and HC PBMC supernatants did not show any statistically significant difference (Figure 6c).

## 4. Discussion

Neuroinflammation is one of the main factors of neurodegenerative diseases pathogenesis including amyotrophic lateral sclerosis (ALS) [6,7,8,13,14,15,16,25]. A key trigger of inflammation process is NLRP3 inflammasome complex, and a few studies illustrate its potential role in ALS-associated neuroinflammation and progression in ALS patients. Post-mortem studies demonstrate the expression of inflammasome components and related cytokines in the CNS [13,25], and studies of biological fluids of ALS patients, the elevated levels of cytokines secreted upon inflammasome activation [26,27,28,29,30]. However, there is a lack of reliable in vivo studies of inflammasome activation and its contribution to neuroinflammation in ALS patients. Given that CNS cells and tissues are unavailable for in vivo sampling, and circulating cytokines do not always reliably indicate the current inflammation status (due to their short half-life, low blood concentrations, and inhibition by the other plasma and cerebrospinal fluid proteins), the immune cells of blood seem to be a worthy alternative for assessing various parameters of inflammation and inflammasome activation. Peripheral blood mononuclear cells (PBMCs) are a mixture of immune cells populations isolated from blood using conventional methods, such as density gradient centrifugation. As a result of isolation, populations of red blood cells, platelets and granulocytes are eliminated and T-lymphocytes and monocytes/macrophages populations are enriched. Studies of neurodegenerative diseases confirm a close relationship between neuroinflammation and inflammation in the periphery [35,36,37,38], and the immune profile of PBMCs reflects the inflammation status and activation of inflammasomes [55].

In our pilot methodological study preceding a planned study of inflammasome-associated biomarkers of neuroinflammation on a larger cohort of ALS patients, we aimed to determine the optimal conditions for handling PBMCs when analyzing inflammasome components expression which is the first essential step of the inflammasome activation pathway. This was motivated by the contradictory data we observed between different trials on the expression of NLRP3 inflammasome components and inflammasome activation performed in PBMCs of patients with neurodegenerative diseases (ALS, Alzheimer disease and Parkinson disease) [44,45,46,47,48,49,50,51,52,53,54,55,62] (Table 1). These discrepancies are likely due to the differences in cell handling conditions, including the use of either non-cultivated or cultivated PBMCs, as well as to the treatment of PBMCs with exogenous stimuli. We also found that few studies have examined inflammasome in human PBMCs in ALS patients.

The first essential step of the two-step process of inflammasome activation is mRNA expression of inflammasome complex components and inflammation-related proteins. So, we compared their mRNA expression levels in PBMCs that were isolated and either subjected (cultivated) or not subjected (non-cultivated) to 21–24 h cultivation. For most genes analyzed, mRNA expression levels were higher in non-cultivated PBMCs than in cultivated PBMCs (Figure 1, Supplementary Table S2). Looking ahead, we have not observed significant differences between expression levels of ALS and HC non-cultivated PBMCs (Supplementary Table S2). Therefore, the elevated expression levels of NLRP3 and cytokines in non-cultivated PBMCs are apparently not an intrinsic feature of ALS PBMCs. Rather they are a general feature of PBMCs independent of ALS. We propose two hypotheses that could explain this effect. First, the expression profile could be shifted because of cultivation-induced change in PBMC subset composition expressed in a loss of some cells and enrichment of specific cellular fractions. Secondly, various factors associated with PBMC isolation procedures (delayed PBMC isolation after blood collection, removal of the cells from their natural environment, use of Ficoll) could make PBMCs responsive for stress [76,77,78,79,80,81,82,83]. As a consequence, the cells may develop stress response and get early activation profile characterized by the higher expression of some cytokines, including TNFα, IL-1β, and IL-6 [84]. A 24 h resting of the cells before performing analysis may help to restore them to their initial state [85] including expression levels of stress-responsive genes. In our experiment, we added the period of overnight resting for cultivated PBMCs. However, it should be kept in mind that 24 h and longer cultivation of PBMCs may diverge their expression profile from their original in vivo state [86]. Additionally, cultivation may introduce the other factors that influence expression profiles, such as changes in cell fraction composition mentioned above, impact of media serum, and changes in RNA stability [81,87,88].

To validate these hypotheses, additional studies should be performed. However, deep detailed analysis of cultivation-induced difference in mRNA expression levels was beyond the scope of our study, and we were mainly interested if the observed effect on mRNA expression will be translated to the protein level. Interestingly, our subsequent studies of protein expression demonstrated equal protein levels of all the inflammasome components, NLRP3, ASC and procaspase-1, between non-cultivated and cultivated PBMCs, as well as between ALS and HC groups. The divergence of the mRNA and protein expression levels is not surprising, and has been observed in many studies associating it with a potential role of processes downstream of transcription that regulate protein abundancy [89,90]. In ALS mouse models and patient studies the discrepancy between mRNA and protein levels has also been found for ASC, IL-1β and IL-18 [13,75]. Surprisingly, PBMC cultivation (in both ALS and HC groups) seemed to exert an effect on the higher accumulation of IL-1β protein, which increased 2–3 fold in comparison to non-cultivated PBMCs (Supplementary Table S3, Supplementary Figure S2). This points to some factor of cultivation that favors the accumulation of IL-1β protein in PBMCs, regardless of its mRNA expression level that was an order of magnitude lower in cultivated PBMCs in comparison to non-cultivated. Probably, this effect may be due to differences at the post-transcriptional level, in particular the rate of IL-1β degradation on the proteasome and its post-translational modifications. To sum up, the results of our pilot study point out that regardless of the large differences in mRNA expression levels of inflammasome components and inflammation-related cytokines in non-cultivated and cultivated PBMCs, they are not necessarily translated to the protein level, which should be kept in mind in studies of inflammasome expression and activation in PBMC-based models.

In most of the studies of Alzheimer disease, Parkinson disease and ALS that we analyzed, cultivated PBMCs were stimulated with exogenous stimuli in order to induce inflammasome components expression necessary for further activation of inflammasome complex. Apparently, immune cells of patients with neurodegenerative diseases are already predisposed to expressing a pro-inflammatory profile. However, under cultivation conditions, they are deprived of natural environmental cues and CNS factors which may be necessary to trigger the peripheral immune system and stimulate their abnormal proinflammatory activity [91]. This justifies the use of exogenous stimuli that can make the signs of proinflammatory activity, including inflammasome activation, more pronounced and susceptible for detection by specific methods. To this end, PBMCs are treated with bacterial lipopolysaccharide (LPS), a widely used priming signal for in vitro NLRP3 inflammasome activation [73,92], or with aggregation-prone proteins/peptides (e.g., amyloid-β peptide, α-synuclein) having pathological roles in neurodegenerative diseases which have a similar effect to LPS [46,93,94,95]. However, using them may shift the focus from discovering the intrinsic features of pathological PBMCs to studying the effects of these stimuli. Moreover, inflammasome expression and activation may occur in the absence of such stimuli, as evidenced from the studies analyzed by us (Table 2, groups of unstimulated PBMCs) and other studies [74]. This calls into question the necessity of using exogenous stimuli. In our pilot experiment we aimed to study the intrinsic features of ALS PBMCs, assuming that additional stimuli would complicate the interpretation of the experimental data. Therefore, we mostly analyzed unstimulated cultivated PBMCs and detected significant expression levels of all the components of the NLRP3 inflammasome complex and inflammasome-activated cytokines in them. However, for some ALS patients and Healthy controls, we have included LPS-stimulated PBMCs, first of all, to investigate the effect of LPS on mRNA expression of inflammasome components and inflammation-related proteins and, secondly, to find out if it can influence the ability to detect differences between the ALS and HC groups. Regarding the first point, we found that in the presence of LPS mRNA expression of cytokine genes was increased: 5–6 fold for IL-1β (which was not however confirmed on a protein level in Western blot) and IL-6, and 1,7 fold for IL-18, which was expected. However, we did not observe the expected elevation of NLRP3 expression level, which may be due to the necessity of a second activating signal for that, missing the peak of mRNA expression, or insufficient incubation time with LPS for this particular gene. For the other gene expression levels (pro-caspase-1, GSDMD) we have not observed the effects of LPS. These genes are constitutively expressed in immune cells [64,65,96]. Likely, their expression levels are sufficient, so LPS treatment does not have a significant effect on them. Speaking of the second point concerning ALS vs. HC differences, LPS treatment of PBMCs has led to the same observations as in LPS-untreated PBMCs: mRNA and protein expression levels were not different between ALS and HC (with one exception mentioned below). To sum up, in inflammasome studies, the necessity of LPS stimulation of cultivated PBMCs is questioned, as all inflammasome components and inflammasome-associated cytokines are expressed at significant levels in LPS-unstimulated cells. To add up in our study, the presence of the LPS signal did not influence the observations concerning ALS effect.

In our study, the only ALS vs. HC difference was observed for mRNA expression level of NLRP3 sensor in cultivated cells only (merged LPS− and LPS+ group, 1,4-fold increase in ALS patients, Figure 3, Supplementary Table S2). This effect is too weak to draw far-reaching conclusions, especially considering that it was not confirmed on the protein level. However, it could be kept in mind in our future inflammasome-associated biomarker studies on a larger cohort of ALS patients given that other studies reported a similar effect for NLRP3 mRNA expression level in the blood [12] and in non-cultivated monocytes of ALS patients [55].

Some ALS studies detect low IL-1β levels in sera/plasma of patients, which are higher than in Healthy controls [28,29,97,98], but in our pilot experiment secreted IL-1β was beyond detection level in plasma of both ALS and HC, despite evidence of IL-1β mRNA and protein expression in PBMCs. This finding is in line with the other ALS studies by Italiani et al. and Zhao et al. [26,55], and could be the consequence of very low abundance of IL-1β in peripheral blood [71], which makes it difficult to detect with conventional assays such as ELISA. Meanwhile, we have detected IL-1β in the supernatants of cultivated PBMCs, and in LPS-stimulated PBMCs (both ALS and HC) its level was higher; no differences were found between ALS and HC groups. The ability to detect significant levels of secreted IL-1β in the supernatants of cultivated PBMCs accompanied by the inability to detect it in plasma by the same assay is explained by the fact that dilution factor of plasma samples analyzed in ELISA is approximately 1000 times higher than of cultured PBMC supernatants and by the above mentioned low concentrations of IL-1β in peripheral blood. Thus, though in comparison to non-cultivated PBMCs in cultivated PBMCs the level of mRNA expression of IL-1β was lower, protein expression was higher, and secreted cytokine could be detected in the supernatants of these cells but not in plasma of ALS patients and controls.

Secreted IL-18, on the contrary to IL-1β, was not detected in cultured supernatants. This is not that surprising given that in our Real-time PCR experiments cultivated PBMC mRNA expression level of IL-18 was much lower than of IL-1β (Ct~28–30 cycles vs. Ct~19–23 cycles), and that IL-18 protein expression was beyond the limit of detection. Meanwhile, unlike IL-1β, IL-18 was detected in both ALS patients and HC plasma, with no difference between the groups (Figure 6). This is in accordance with the data on the higher concentrations of IL-18 circulating in healthy human plasma determined by the higher concentration required for its activity (10–20 ng/mL and even higher in comparison to IL-1β requiring low nanograms up to picograms per milliliter) [71,99]. A number of studies report higher IL-18 levels in ALS patients plasma and cerebrospinal fluid, in comparison to Healthy controls [26,30,97]; however, in our study we did not observe this effect, probably due to a small sample size.

The focus of our study was the investigation of the first step of inflammasome activation, namely induction of mRNA expression of its complex proteins and IL-1β and IL-18 and subsequent accumulation of corresponding proteins. The second inflammasome activation step is its complex assembly, with subsequent auto-cleavage of caspase-1 and processing and secretion of IL-1β, IL-18 and GSDMD which was not analyzed specifically in our study. The cleaved forms of caspase-1 and IL-1β and IL-18 proteins which could evidence inflammasome activation and could be theoretically detected in Western blot, were not detected in our study, apparently due to the insufficient antibody sensitivity for mature protein forms in our set of experimental conditions. Also, conventional ELISA methods used by us do not distinguish between precursor and cleaved forms of IL-1β and IL-18 cytokines. Thus, we cannot provide the strict evidence for inflammasome activation in ALS PBMCs in our study but plan to apply additional methods in our future studies to be able to do that.

Our study has several limitations. First, it has a small sample size because we focused primarily on analyzing the differences between non-cultivated and cultivated PBMCs isolated from one subject and processed in parallel. We plan to continue our research on a larger cohort of ALS patients, focusing on either non-cultivated or cultivated PBMCs and include a correlation analysis of inflammasome components and cytokines expression data with the clinical parameters of ALS patients, which we also have not performed here. Of note, all ALS patients received riluzole as disease-modifying therapy, and thus its effect on inflammatory signaling could not be assessed within this cohort. Riluzole is primarily an anti-excitotoxic agent which inhibits glutamate release and voltage-gated sodium-channel blockade [100]. Its secondary anti-inflammatory action has been reported in preclinical models, i.e., attenuation of pro-inflammatory activity and a shift toward M2 microglia/macrophage polarization after rodent spinal-cord injury was shown by Wu et al. [101]. However, there is no direct evidence that riluzole modulates the NLRP3 inflammasome or PBMCs. We therefore do not overstate this point: should any mild anti-inflammatory effect exist, it would be expected to attenuate rather than exaggerate ALS vs. HC differences, and so it cannot account for the conservative, correction-based findings we report. We also have not studied in a detailed way the reasons for difference in mRNA expression profiles between non-cultivated and cultivated PBMCs; it could be done in future studies by analyzing PBMC cell fractions, activation state, adherence properties, and viability prior and post-culture by using flow cytometry. Additionally, by conventional methods like Western blot and ELISA, we were unable to detect obvious signs of inflammasome activation, particularly cleaved forms of caspase-1, IL-1β, and IL-18 proteins. The main focus of our study was to investigate mRNA expression and protein accumulation of NLRP3 inflammasome components and IL-1β and IL-18, the initial stages of NLRP3 inflammasome activation pathway. However, in our future studies, inflammasome activation may be evaluated by Western blot using antibodies with higher sensitivity to mature protein forms or by the other, more specific and powerful assays measuring caspase-1 activity, ASC specks formation and cleavage of mature cytokines and GSDMD. The issue of undetectable IL-1β in plasma can also be resolved by using assays with higher sensitivity.

## 5. Conclusions

Thus, our pilot study on PBMCs of ALS patients and Healthy controls revealed that mRNA expression level of most analyzed inflammasome components and inflammation-related proteins in non-cultivated PBMCs were significantly higher than in PBMCs subjected to 24 h cultivation, which, however, was not translated into protein level. In cultivated PBMCs, as expected, LPS stimulation elevated the expression levels of pro-inflammatory cytokines but did not influence our ability to observe ALS-specific effect: in both LPS− and LPS+ cultivated, as well as in non-cultivated PBMCs, no differences between ALS patients and Healthy controls were found, with the exception of slightly elevated mRNA expression of NLRP3 in cultivated ALS PBMCs. Thereby, LPS stimulation appears to be an optional requirement for such studies. In general, in our study PBMC cultivation had an impact on the expression of inflammasome components and inflammation-related proteins only on mRNA, but not protein level, which suggests that similar studies could be performed in either non-cultivated or cultivated PBMCs. However, possible effects of cell handling conditions must be considered when applying the study results to the broader picture of ALS pathogenesis as well as the other neurodegenerative and non-neural disorders.

## Figures and Tables

**Table 1:** Clinical characteristics of study participants.

Variable	ALS (*n* = 23)	HC (*n* = 20)	*p*-Value
Gender, male/female	9/14	9/11	0.76 ^2^
Age (years) ^1^	53.0 [51; 61]	54.5 [5.25; 50.59]	0.98 ^3^
Disease Duration (months)	15 [9; 28]	N.A.	N.A.
ALSFRS-R	40 [37; 42]	N.A.	N.A.
Progression rate	0.56 [0.4; 0.8]	N.A.	N.A.

^1^ Values hereinafter are expressed as median [Q1, Q3]; ^2^ Fisher’s exact test; ^3^ Mann–Whitney U test; ALS—patients with ALS; HC—Healthy controls; N.A.—not available.

**Table 2:** PBMC-based studies of NLRP3 inflammasome expression/activation in patients with neurodegenerative diseases.

# of Study	Reference	Disease	Assays	PBMCs Handling Conditions	Results
1	[62]	AD	Human Inflammasomes RT2 Profiler PCR Array and RT-PCR; WB, confocal microscropy and flow cytometry; ELISA (supernatants)	After 2 h cultivation, non-adherent PBMCs were washed out. Adherent monocytes: (1) unst, (2) LPS (2 μg/mL, 2 h), (3) Aβ42 (10 μg/mL, 24 h), (4) LPS (2 μg/mL, 2 h) + Aβ42 (10 μg/mL, 24 h).	AD monocytes compared to HC: significant difference only in (4) group: mRNA: ↑ NLRP3, ASC, caspase-1, IL-1β, IL-18 WB: ↑ NLRP3 Confocal microscopy: co-localization of NLRP3, ASC and caspase-1 Flow cytometry: ↑ of % monocytes NLRP3+/caspase-1+ and NLRP3+/ASC+ Supernatants: ↑ IL-1β, IL-18
2	[50]	AD	RT-PCR or RNA-sequencing; WB; ELISA (plasma)	PBMC Non-Cult	AD PBMCs compared to HC: mRNA (RNA-seq): ↑ caspase-1 mRNA (RT-PCR): ↑ NLRP3, caspase-1, IL-1β, GSDMD WB: ↑ NLRP3, cleaved caspase-1, cleaved IL-1 β, cleaved GSDMD Plasma: ↑ IL-1β
3	[44]	AD	AMNIS FlowSight; ELISA (supernatants)	PBMC Cult: (1) unst, (2) LPS (1 μg/mL, 2 h) + Aβ42 (10 μg/mL, 22 h)	(2) AD PBMCs compared to (1) AD PBMCs: AMNIS FlowSight: NLRP3 and ASC co-localization Supernatants: ↑ IL-1β, IL-18, cleaved caspase-1
4	[51]	PD	ELISA (supernatants)	PBMC Cult: (1) unst, (2) LPS (1 µg/mL, 24 h)	PD PBMC supernatants compared to HC: equal levels of IL-1β (2) ↑ IL-1β
5	[52]	PD	ELISA (supernatants)	PBMC Cult: (1) unst, (2) LPS (10 µg/mL, 24 h)	PD PBMCs compared to HC: ↓ IL-1β in (1), (2)
6	[45]	PD	RT-PCR; ELISA (supernatants)	PBMC Cult: (1) unst, (2) LPS (1 ng/mL, o/n)	PD PBMCs compared to HC in both (1) and (2) groups: mRNA: ↑ IL-1β Supernatants: ↑ IL-1β; in (2) LPS elevated IL-1β levels 25–30-fold
7	[46]	PD (PD model on HC PBMCs)	RT-PCR; WB; ELISA (supernatants)	After cultivation, non-adherent PBMCs were washed out. Adherent monocytes Cult: (1) unst, (2) αSyn-M (1 μM; 2, 6, 18 h), (3) α-synF (40 nM; 2, 6, 18 h), (4) LPS (1 µg/mL; 2, 6, 18 h)	mRNA: ↑ NLRP3 in (3) compared to (1); ↑ IL-1β in (2), (3), (4) compared to (1) WB: ↑ pro-IL-1β and cleaved IL-1β in both (3) and (4) compared to (1) and (2); ↑ caspase-1 (mature) in (3) compared to (1) ELISA: ↑ IL-1β in (3), (4) compared to (1), (2)
8	[47]	PD	RT-PCR; ELISA and Multiplexed High-Throughput Serological Assay (supernatants)	PBMC Cult: (1) unst, (2) LPS (1 ng/mL, 24 h), (3) α-synM (2 nmol/mL, 24 h), (4) α-synF (2 nmol/mL, 24 h)	PD and HC PBMCs had equal effects in (2), (3), (4) groups: mRNA: ↑ IL-1β Supernatants: ↑ IL-1β, IL-18
9	[53]	PD	RT-PCR; WB; Meso Scale Discovery (plasma)	Pellets of cryopreserved PBMCs, Non-Cult	PD PBMCs compared to HC: mRNA: ↑ NLRP3, ASC, caspase-1; equal levels of IL-1β Proteins: ↑ NLRP3, active caspase-1, mature IL-1β Plasma: ↑ IL-1β
10	[48]	PD	AMNIS FlowSight; ELISA (supernatants)	PBMC Cult: (1) unst, (2) LPS (1 μg/mL, 24 h), (3) αSyn-M (5 μM, 24 h), (4) α-synF (10 nM, 24 h), (5) LPS (1 μg/mL, 2 h) + αSynM (5 μM, 24 h), (6) LPS (1 μg/mL, 2 h) + αSynF (10 nM, 24 h)	PD PBMCs compared to HC—similar effects: ASC speck: not detected in (1)–(6) Supernatants: equal cleaved caspase-1 and IL-18 levels in (1)–(6); ↑ IL-1β in (5) and (6) groups
11	[54]	PD	RT-PCR; Immunochemistry; ELISA (plasma)	PBMC Non-Cult and frozen or fixed (immunochemistry)	PD PBMCs compared to HC: mRNA: ↑ NLRP3, casp-1 Immunochemistry: ↑ NLRP3 Plasma: equal levels of IL-1β, IL-18
12	[49]	PD	Multiplex ELISA (supernatants)	PBMC Cult: (1) unst, (2) α-synM (0.5 µM, 24 h), (3) α-synO (0.5 µM, 24 h)	PD PBMC supernatants: equal IL-1β, IL-18 levels in (1), (2), (3) HC PBMC supernatants: ↑ IL-1β, IL-18 in (2), (3) in comparison to (1) PD PBMCs supernatants compared to HC: ↑ IL-1β, IL-18 in (1), equal IL-1β, IL-18 levels in (2), (3)
13	[55]	ALS (sporadic)	RNA-sequencing and RT-PCR; ELISA (serum)	Monocytes negatively selected by Monocyte Isolation Kit, Non-Cult	ALS monocytes compared to HC: mRNA: ↑ IL-1β, NLRP3 Serum: equal background level of IL-1β

↑ and ↓ indicate increase or decrease of the level of the designated parameter. Aβ42—1–42 amyloid-β peptide oligomer; ALS—Amyotrophic Lateral Sclerosis; AD—Alzheimer disease; Cult—cultivated; HC—Healthy controls; LPS—lipopolysaccharide; Non-Cult—non-cultivated; PD—Parkinson disease; Unst—unstimulated; WB—Western blot; αSyn-M—α-synuclein monomers; αSyn-O—α-synuclein oligomers; αSyn-F—α-synuclein fibrils.

## Data Availability

Data is contained within the article.

## Supplementary Materials

The following supporting information can be downloaded at: https://www.mdpi.com/article/10.3390/neurosci7040084/s1, Table S1: Primers used for Real-time PCR; Table S2: Statistical comparisons of mRNA expression levels of genes encoding NLRP3 inflammasome components and inflammation-related proteins in PBMCs of ALS patients and Healthy controls; Table S3: Statistical comparisons of relative protein expression levels of NLRP3 inflammasome components (NLRP3, ASC, pro-caspase-1) and pro-IL-1β; Table S4: Statistical comparisons of IL-18 plasma concentration levels and IL-1β levels in cultivated PBMC supernatants; Figure S1: Antibodies validation by Western blot of the lysates of HEK293T cells untransfected (lane 1) or transfected with 1× (lane 2) or 2× dose (lane 3) of the plasmids encoding proteins of interest; Figure S2: Protein expression levels of NLRP3 inflammasome components and IL-1β in non-cultivated and cultivated PBMCs of ALS patients and Healthy controls.

## Abbreviations

The following abbreviations are used in this manuscript:

Aβ42	1–42 amyloid-β peptide oligomer
αSyn	α-synuclein
AD	Alzheimer Disease
ALS	Amyotrophic lateral sclerosis
ASC	Apoptosis-associated speck-like protein containing a caspase recruitment domain (CARD)
CNS	Central Nervous System
Cult	Cultivated
DAMP	Damage-associated molecular pattern
ELISA	Enzyme-linked immunosorbent assay
FBS	Fetal Bovine Serum
HC	Healthy Controls
LPS	Lipopolysaccharide
Non-Cult	Non-cultivated
NLRP3	Nucleotide-binding domain, leucine-rich–containing family, pyrin domain–containing-3
PAMP	Pathogen-associated Molecular Pattern
PBMC	Peripheral blood mononuclear Cells
PD	Parkinson Disease
PRR	Pattern Recognition Receptors
Unst	Unstimulated
