# Supplementary material for: NLRP3 Inflammasome and Inflammation-Related Proteins Expression Characterized in Non-Cultivated Versus Cultivated Peripheral Blood Cells of Patients with Amyotrophic Lateral Sclerosis
Source: NeuroSci. 2026 Jul 23;7(4):84. doi: 10.3390/neurosci7040084 (PMC13516774; doi:10.3390/neurosci7040084)
Supplement: Supplementary file 1 [file neurosci-07-00084-s001.zip › neurosci-4320202-supplementary.pdf]

Supplementary Table S1. Primers used for Real-time PCR.

Gene	Forward Primer Sequence (5′-3′)	Reverse Primer Sequence (5′-3′)
ASC	CCAGGCCTGCACTTTATAGAC	ACTGCTCATCCGTCAGGA
Caspase-1	CAGGACAACCCAGCTATG	GGATAAATCTCTGCCGACTT
GSDMD	CTGACCATGCTGAGTGAAAC	GGCACTCTGCTCCAAGA
NLRP3	CTTCAGGTGTTGGAATTAGACA	CAGCACTTCACAGAACATCA
IL1-β	GTGTTCTCCATGTCCTTTGT	TTGGGATCTACACTCTCCAG
IL-6	CATGTGTGAAAGCAGCAAAG	CAGGCAAGTCTCCTCATTG
IL-18	GAGGTATGGCTGTAACTATC	CAGGAGGATTCATTTCCTTA
IL-18BP	TAAGCAGTGTCCAGCATTG	ATTGCCCAGCCAGTAGA
RPL13A	ACCGTGCGAGGTATGCT	CCCAGATAGGCAAACTTTCTTGTA

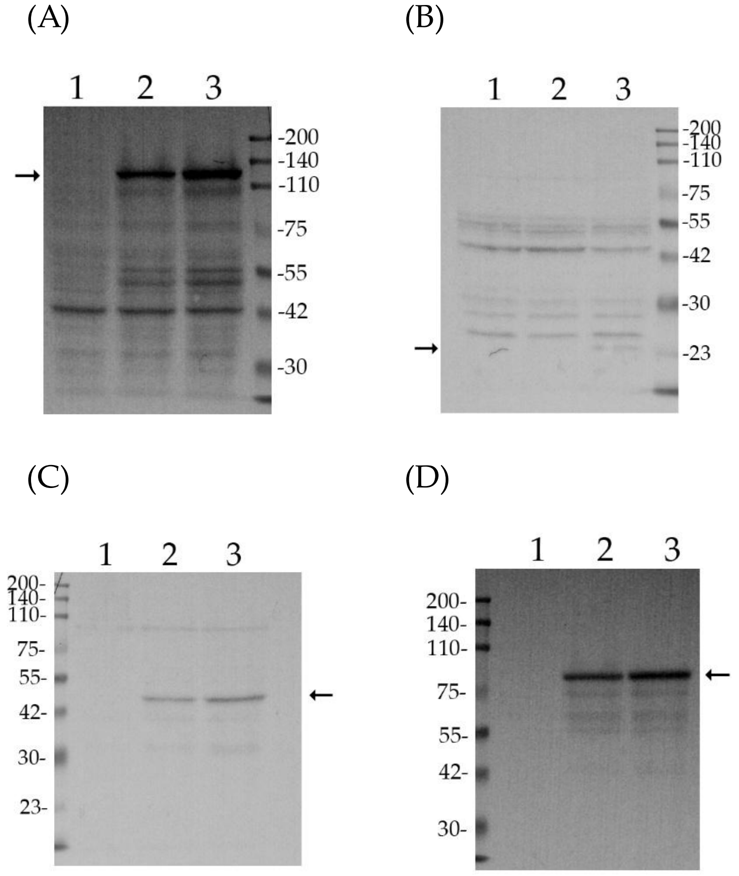

Supplementary Figure S1. Antibodies validation by Western blot of the lysates of HEK293T cells untransfected (lane 1) or transfected with 1x (lane 2) or 2x dose (lane 3) of the plasmids encoding proteins of interest. Blots were stained with anti-NLRP3 (A), anti-ASC (B), anti-caspase-1 (C) or anti-IL-1b(D) antibodies. The positions of proteins of interest are marked with the arrows.

Validation of antibodies was performed on the lysates of HEK293T cells which are NLRP3-, ASC-, procaspase-1- and IL-1β-negative. The cells were transfected with commercial plasmids (all from Addgene): (A) pEGFP-C2-NLRP3 (#73955) encoding EGFP-tagged NLRP3 (EGFP-NLRP3 Mw=130 kDa, and expected NLRP3 Mw=110 kDa); (B) pCI ASC-HA (#41553) encoding HA-tagged ASC (ASC-HA Mw=23 kDa, and expected ASC Mw=22 kDa); (C) pCI casp1 (#41552) encoding Myc-tagged pro-caspase-1 (Casp-1-Myc Mw=46 kDa, and expected casp-1 Mw=45 kDa); (D) pLV-mTurquoise2-IL-1beta-mNeonGreen (#166783) encoding IL-1β tagged with mTurquoise2 and mNeonGreen (mTurquoise2-IL-1beta-mNeonGreen Mw=85 kDa, and expected IL-1β Mw=31 kDa). HEK293T cells were transfected by GenJect 39 (Molecta, Russia) according to manufactures instructions. Next day cells were lysed in Laemmli buffer and subjected to 8 or 10% PAAG and blotted onto nitrocellulose membrane. Membranes were blocked in PBS-T buffer with addition of skim milk (PBS, 0,05% Tween 20, 5% skim milk) for 2 h followed by overnight incubation with antibodies specific for inflammasome components and IL-1β: NLRP3 (AG-20B-0014-C100), ASC (AG-25B-0006-C100), caspase-1 (AG-20B-

0048-C100) (all from Adipogen, USA), IL-1β (ab216995, Abcam, USA) diluted in blocking buffer containing PBS with 0,05% Tween-20 and 5% skim milk or BSA in concentrations recommended by manufacturers. The bands corresponding to the expected sizes of proteins of interest were only detected in the transfected cells (lanes 2 and 3) and were identified as specific; the other bands were considered non-specific and omitted from the analysis in subsequent Western blots with PBMC lysates.

Supplementary Table S2. Statistical comparisons of mRNA expression levels of genes encoding NLRP3 inflammasome components and inflammation-related proteins in PBMCs of ALS patients and Healthy controls. The subgroups being compared are listed in the left column of the table, p-values were calculated by Mann-Whitney U tests, and for multiple testing, p-values were adjusted within each family of comparisons (the panel of genes for a given contrast) with the Benjamini–Hochberg false-discovery-rate procedure (reported as q); p-values and an adjusted $q < 0.05$ was considered statistically significant and are indicated in red; effect sizes are reported as the rank-biserial correlation; 95% confidence intervals for the difference in medians were estimated with the Hodges–Lehmann method.

Compared PBMC subgroups	NLRP3	ASC	Procaspase-1	Pro-IL-1β	Pro-IL-18	IL-18BP	GSDMD	IL-6
**Non-Cult (ALS and HC)**	0,05195 [0,03004; 0,08105], n=33	0,002762 [0,002074; 0,003042], n=33	0,01621 [0,01107; 0,02234], n=33	0,9794 [0,4737; 1,678], n=33	0,005839 [0,002333; 0,009539], n=33	0,003048 [0,002223; 0,003674], n=30	0,0001916 [8,793e-005; 0,0003530], n=30	0,1015 [0,005934; 0,1466], n=30
**Cult[1] (ALS and HC)**	0,001193 [0,001062; 0,001782], n=38	0,001128 [0,0009368; 0,001416], n=38	0,01226 [0,008759; 0,01527], n=38	0,1182 [0,04450; 0,3187], n=38	0,0004340 [0,0002680; 0,0005412], n=38	0,004206 [0,003529; 0,006976], n=35	0,0007034 [0,0003312; 0,001042], n=35	0,02110 [0,008995; 0,05646], n=35
p-value (raw)	<0,0001	<0,0001	0,0044	<0,0001	<0,0001	<0,0001	<0,0001	0,0139
q (BH–FDR)	**<0,0001**	**<0,0001**	**0,0050**	**<0,0001**	**<0,0001**	**<0,0001**	**<0,0001**	**0,0139**
Effect size, r	+1,00	+0,96	+0,39	+0,78	+0,96	-0,59	-0,72	+0,36
Median diff [95% CI]	0,0506 [0,0475; 0,0562]	0,00144 [0,00132; 0,0016]	0,00372 [0,00259; 0,00534]	0,817 [0,702; 0,914]	0,00538 [0,00493; 0,00583]	-0,00172 [-0,00222; -0,0013]	-0,000444 [-0,000524; -0,000348]	0,0586 [0,0458; 0,0729]
**Non-Cult (ALS and HC)**	0,05195 [0,03004; 0,08105], n=33	0,002762 [0,002074; 0,003042], n=33	0,01621 [0,01107; 0,02234], n=33	0,9794 [0,4737; 1,678], n=33	0,005839 [0,002333; 0,009539], n=33	0,003048 [0,002223; 0,003674], n=30	0,0001916 [8,793e-005; 0,0003530], n=30	0,1015 [0,005934; 0,1466], n=30
**Cult LPS-(ALS and HC)**	0,001197 [0,001054; 0,001728], n=23	0,001183 [0,0009811; 0,001428], n=23	0,01392 [0,008892; 0,01698], n=23	0,06121 [0,03228; 0,1210], n=23	0,0003221 [0,0002369; 0,0004630], n=23	0,005627 [0,004148; 0,007581], n=23	0,0007083 [0,0004106; 0,001069], n=23	0,009843 [0,004280; 0,02149], n=23
p-value (raw)	<0,0001	<0,0001	0,0633	<0,0001	<0,0001	<0,0001	<0,0001	0,0054
q (BH–FDR)	**<0,0001**	**<0,0001**	0,0633	**<0,0001**	**<0,0001**	**<0,0001**	**<0,0001**	**0,0062**
Effect size, r	+1,00	+0,94	+0,30	+0,91	+0,98	-0,87	-0,80	+0,45
Median diff [95% CI]	0,0506 [0,047; 0,0585]	0,00135 [0,00117; 0,00156]	0,00312 [0,00165; 0,00472]	0,893 [0,793; 1,05]	0,00552 [0,00501; 0,00598]	-0,00253 [-0,00318; -0,00208]	-0,000464 [-0,000567; -0,000372]	0,072 [0,0578; 0,0998]
**Non-Cult (ALS and HC)**	0,05195 [0,03004; 0,08105], n=33	0,002762 [0,002074; 0,003042], n=33	0,01621 [0,01107; 0,02234], n=33	0,9794 [0,4737; 1,678], n=33	0,005839 [0,002333; 0,009539], n=33	0,003048 [0,002223; 0,003674], n=30	0,0001916 [8,793e-005; 0,0003530], n=30	0,1015 [0,005934; 0,1466], n=30
**Cult LPS+(ALS and HC)**	0,001188 [0,001114; 0,001971], n=15	0,001016 [0,0006717; 0,001212], n=15	0,01062 [0,008669; 0,01263], n=15	0,3143 [0,1282; 0,6721], n=15	0,0005430 [0,0004310; 0,0008118], n=15	0,002579 [0,002308; 0,003891], n=12	0,0005849 [0,0002578; 0,0009531], n=12	0,06375 [0,04446; 0,08421], n=12
p-value (raw)	<0,0001	<0,0001	0,0027	0,0011	<0,0001	0,7594	0,0047	0,3805
q (BH–FDR)	**<0,0001**	**<0,0001**	**0,0043**	**0,0022**	**<0,0001**	0,7594	**0,0063**	0,4348
Effect size, r	+1,00	+0,99	+0,55	+0,60	+0,92	-0,06	-0,57	+0,18
Median diff [95% CI]	0,0505 [0,0466; 0,0606]	0,00157 [0,00141; 0,00178]	0,00522 [0,00317; 0,00754]	0,63 [0,463; 0,816]	0,00521 [0,00451; 0,00579]	-7,03e-05 [-0,000382; 0,000148]	-0,000362 [-0,0005; -0,000206]	0,0269 [-0,00266; 0,052]
**Non-Cult ALS**	0,07476 [0,03475; 0,09606], n=19	0,002553 [0,002003; 0,003133], n=19	0,01987 [0,01201; 0,02235], n=19	1,240 [0,7440; 1,682], n=19	0,006059 [0,004508; 0,009121], n=19	0,002366 [0,002213; 0,003587], n=16	0,0001838 [0,0001085; 0,0003280], n=16	0,08911 [0,02187; 0,1421], n=16
**Cult ALS**	0,001397 [0,001151; 0,002223], n=23	0,001169 [0,001016; 0,001428], n=23	0,01062 [0,008669; 0,01263], n=23	0,1272 [0,06515; 0,3914], n=23	0,0004917 [0,0003221; 0,0005661], n=23	0,004918 [0,003562; 0,007684], n=20	0,0007537 [0,0004401; 0,001028], n=20	0,02205 [0,007451; 0,06827], n=20
p-value (raw)	<0,0001	<0,0001	0,0034	<0,0001	<0,0001	0,0003	<0,0001	0,0416
q (BH–FDR)	**<0,0001**	**<0,0001**	**0,0039**	**<0,0001**	**<0,0001**	**0,0004**	**<0,0001**	**0,0416**
Effect size, r	+1,00	+0,95	+0,53	+0,82	+0,95	-0,72	-0,80	+0,40

Compared PBMC subgroups	NLRP3	ASC	Procaspase-1	Pro-IL-1β	Pro-IL-18	IL-18BP	GSDMD	IL-6
Median diff [95% CI]	0,0728 [0,0612; 0,077]	0,00138 [0,0011; 0,00157]	0,00537 [0,0034; 0,00723]	0,935 [0,815; 1,16]	0,00553 [0,0052; 0,00586]	-0,00236 [-0,0032; -0,00163]	-0,000523 [-0,000631; -0,00043]	0,0563 [0,0409; 0,0691]
**Non-Cult ALS**	0,07476 [0,03475; 0,09606], n=19	0,002553 [0,002003; 0,003133], n=19	0,01987 [0,01201; 0,02235], n=19	1,240 [0,7440; 1,682], n=19	0,006059 [0,004508; 0,009121], n=19	0,002366 [0,002213; 0,003587], n=16	0,0001838 [0,0001085; 0,0003280], n=16	0,08911 [0,02187; 0,1421], n=16
**Cult LPS- ALS**	0,001460 [0,001126; 0,002084], n=13	0,001183 [0,001083; 0,001538], n=13	0,01395 [0,009853; 0,01688], n=13	0,07322 [0,04129; 0,1557], n=13	0,0003509 [0,0002191; 0,0005079], n=13	0,006479 [0,004297; 0,007968], n=13	0,0007124 [0,0004659; 0,001068], n=13	0,01059 [0,004514; 0,03376], n=13
p-value (raw)	<0,0001	<0,0001	0,0551	<0,0001	<0,0001	<0,0001	<0,0001	0,0226
q (BH–FDR)	**<0,0001**	**<0,0001**	0,0551	**<0,0001**	**<0,0001**	**<0,0001**	**<0,0001**	**0,0258**
Effect size, r	+1,00	+0,94	+0,41	+0,94	+0,98	-0,89	-0,93	+0,50
Median diff [95% CI]	0,0728 [0,0609; 0,0785]	0,00132 [0,000991; 0,00156]	0,00405 [0,00259; 0,00619]	1,1 [0,899; 1,35]	0,00564 [0,00526; 0,00607]	-0,00316 [-0,00419; -0,00241]	-0,000527 [-0,000634; -0,000407]	0,0637 [0,0522; 0,0998]
**Non-Cult ALS**	0,07476 [0,03475; 0,09606], n=19	0,002553 [0,002003; 0,003133], n=19	0,01987 [0,01201; 0,02235], n=19	1,240 [0,7440; 1,682], n=19	0,006059 [0,004508; 0,009121], n=19	0,002366 [0,002213; 0,003587], n=16	0,0001838 [0,0001085; 0,0003280], n=16	0,08911 [0,02187; 0,1421], n=16
**Cult LPS+ ALS**	0,001268 [0,001153; 0,002909], n=10	0,001113 [0,0009743; 0,001424], n=10	0,01067 [0,008664; 0,01276], n=10	0,3791 [0,1462; 0,8200], n=10	0,0005546 [0,0004780; 0,001238], n=10	0,002820 [0,002421; 0,007025], n=7	0,0008462 [0,0001974; 0,0009868[, n=7	0,07105 [0,04239; 0,08040], n=7
p-value (raw)	<0,0001	<0,0001	0,0027	0,0041	<0,0001	0,1508	0,0329	0,4515
q (BH–FDR)	**0,0001**	**0,0001**	**0,0053**	**0,0066**	**0,0002**	0,1723	**0,0438**	0,4515
Effect size, r	+1,00	+0,97	+0,69	+0,66	+0,93	-0,39	-0,57	+0,21
Median diff [95% CI]	0,0721 [0,0597; 0,0789]	0,0015 [0,00111; 0,00175]	0,00759 [0,00504; 0,00946]	0,801 [0,577; 1,03]	0,0053 [0,00471; 0,00582]	-0,000494 [-0,00145; -0,000155]	-0,000521 [-0,000681; -0,000365]	0,0362 [-0,00266; 0,0554]
**Non-Cult HC**	0,04304 [0,02131; 0,05419], n=14	0,002807 [0,002099; 0,002949], n=14	0,01282 [0,009541; 0,02249], n=14	0,8027 [0,2621; 1,249], n=14	0,005211 [0,001401; 0,01061], n=14	0,003126 [0,002338; 0,003775], n=14	0,0002055 [7,583e-005; 0,0003835], n=14	0,1039 [0,002495; 0,2112], n=14
**Cult HC**	0,001064 [0,0007490; 0,001197], n=15	0,0009543 [0,0007556; 0,001225], n=15	0,01203 [0,008669; 0,01599], n=15	0,08677 [0,03228; 0,2973], n=15	0,0003282 [0,0002369; 0,0004462], n=15	0,004007 [0,002344; 0,005653], n=15	0,0005405 [0,0002678; 0,001069], n=15	0,01527 [0,009163; 0,05065], n=15
p-value (raw)	<0,0001	<0,0001	0,2475	0,0004	<0,0001	0,0471	0,0073	0,1979
q (BH–FDR)	**<0,0001**	**<0,0001**	0,2475	**0,0008**	**<0,0001**	0,0627	**0,0116**	0,2262
Effect size, r	+1,00	+0,94	+0,26	+0,78	+0,99	-0,44	-0,59	+0,29
Median diff [95% CI]	0,0419 [0,04; 0,0469]	0,0016 [0,00144; 0,00179]	0,00222 [0,000412; 0,0041]	0,688 [0,488; 0,811]	0,00483 [0,0035; 0,00722]	-0,000998 [-0,0016; -0,000534]	-0,000264 [-0,000459; -0,000189]	0,0648 [0,013; 0,0954]
**Non-Cult HC**	0,04304 [0,02131; 0,05419], n=14	0,002807 [0,002099; 0,002949], n=14	0,01282 [0,009541; 0,02249], n=14	0,8027 [0,2621; 1,249], n=14	0,005211 [0,001401; 0,01061], n=14	0,003126 [0,002338; 0,003775], n=14	0,0002055 [7,583e-005; 0,0003835], n=14	0,1039 [0,002495; 0,2112], n=14
**Cult LPS- HC**	0,001059 [0,0007338; 0,001240], n=10	0,001154 [0,0009314; 0,001289], n=10	0,01232 [0,008545; 0,01736], n=10	0,05117 [0,02643; 0,1062], n=10	0,0002939 [0,0002335; 0,0004238], n=10	0,004917 [0,003923; 0,006622], n=10	0,0006244 [0,0002545; 0,001106], n=10	0,009787 [0,003634; 0,01612], n=10
p-value (raw)	<0,0001	0,0002	0,4642	0,0003	<0,0001	0,0008	0,0109	0,1354

Compared PBMC subgroups	NLRP3	ASC	Procaspase-1	Pro-IL-1β	Pro-IL-18	IL-18BP	GSDMD	IL-6
q (BH–FDR)	**0,0002**	**0,0005**	0,4642	**0,0005**	**0,0002**	**0,0012**	**0,0145**	0,1547
Effect size, r	+1,00	+0,91	+0,19	+0,90	+1,00	-0,83	-0,63	+0,37
Median diff [95% CI]	0,0419 [0,0399; 0,0471]	0,0015 [0,00117; 0,00168]	0,00206 [-0,000702; 0,00418]	0,713 [0,562; 0,855]	0,00488 [0,00342; 0,00736]	-0,00198 [-0,00254; -0,00152]	-0,000359 [-0,000581; -0,000215]	0,0888 [0,0544; 0,108]
**Non-Cult HC**	0,04304 [0,02131; 0,05419], n=14	0,002807 [0,002099; 0,002949], n=14	0,01282 [0,009541; 0,02249], n=14	0,8027 [0,2621; 1,249], n=14	0,005211 [0,001401; 0,01061], n=14	0,003126 [0,002338; 0,003775], n=14	0,0002055 [7,583e-005; 0,0003835], n=14	0,1039 [0,002495; 0,2112], n=14
**Cult LPS+ HC**	0,001114 [0,0007593; 0,001554], n=5	0,0006717 [0,0006318; 0,0008963], n=5	0,01028 [0,008205; 0,01388], n=5	0,2973 [0,1127; 0,4932], n=5	0,0004310 [0,0002646; 0,0007617], n=5	0,002296 [0,002249; 0,003130], n=5	0,0003312 [0,0002611; 0,0008360], n=5	0,05646 [0,04642; 0,1229], n=5
p-value (raw)	0,0002	0,0014	0,2193	0,0870	0,0003	0,2976	0,1068	0,7539
q (BH–FDR)	**0,0014**	**0,0037**	0,2924	0,1709	**0,0014**	0,3401	0,1709	0,7539
Effect size, r	+1,00	+1,00	+0,40	+0,54	+0,97	+0,34	-0,51	+0,11
Median diff [95% CI]	0,0418 [0,0347; 0,0493]	0,00193 [0,00155; 0,00216]	0,00253 [0,000536; 0,00605]	0,536 [0,206; 0,721]	0,00469 [0,00158; 0,00732]	0,000693 [6,03e-05; 0,000856]	-0,0002 [-0,000272; -0,000148]	0,0206 [-0,0409; 0,0634]
**Non-Cult ALS**	0,07476 [0,03475; 0,09606], n=19	0,002553 [0,002003; 0,003133], n=19	0,01987 [0,01201; 0,02235], n=19	1,240 [0,7440; 1,682], n=19	0,006059 [0,004508; 0,009121], n=19	0,002366 [0,002213; 0,003587], n=16	0,0001838 [0,0001085; 0,0003280], n=16	0,08911 [0,02187; 0,1421], n=16
**Non-Cult HC**	0,04304 [0,02131; 0,05419], n=14	0,002807 [0,002099; 0,002949], n=14	0,01282 [0,009541; 0,02249], n=14	0,8027 [0,2621; 1,249], n=14	0,005211 [0,001401; 0,01061], n=14	0,003126 [0,002338; 0,003775], n=14	0,0002055 [7,583e-005; 0,0003835], n=14	0,1039 [0,002495; 0,2112], n=14
p-value (raw)	0,0302	0,9709	0,2089	0,1502	0,7021	0,4927	1,0000	0,9834
q (BH–FDR)	0,2417	1,0000	0,5570	0,5570	1,0000	0,9854	1,0000	1,0000
Effect size, r	+0,45	+0,01	+0,26	+0,30	+0,08	-0,15	+0,00	+0,01
Median diff [95% CI]	0,0272 [0,0134; 0,038]	6,69e-06 [-0,000233; 0,000218]	0,003 [0,000417; 0,00568]	0,382 [0,0901; 0,655]	0,000566 [-0,000865; 0,00207]	-0,000282 [-0,000749; 0,000109]	1,43e-06 [-4,54e-05; 4,61e-05]	0,000204 [-0,0435; 0,0258]
**Cult ALS**	0,001397 [0,001151; 0,002223], n=23	0,001169 [0,001016; 0,001428], n=23	0,01062 [0,008669; 0,01263], n=23	0,1272 [0,06515; 0,3914], n=23	0,0004917 [0,0003221; 0,0005661], n=23	0,004918 [0,003562; 0,007684], n=20	0,0007537 [0,0004401; 0,001028], n=20	0,02205 [0,007451; 0,06827], n=20
**Cult HC**	0,001064 [0,0007490; 0,001197], n=15	0,0009543 [0,0007556; 0,001225], n=15	0,01203 [0,008669; 0,01599], n=15	0,08677 [0,03228; 0,2973], n=15	0,0003282 [0,0002369; 0,0004462], n=15	0,004007 [0,002344; 0,005653], n=15	0,0005405 [0,0002678; 0,001069], n=15	0,01527 [0,009163; 0,05065], n=15
p-value (raw)	0,0023	0,0470	0,6867	0,1695	0,0732	0,1666	0,2713	0,5157
q (BH–FDR)	**0,0185**	0,1881	0,6867	0,2712	0,1951	0,2712	0,3617	0,5894
Effect size, r	+0,59	+0,39	+0,08	+0,27	+0,35	+0,28	+0,22	+0,13
Median diff [95% CI]	0,000422 [0,000315; 0,000559]	0,000215 [0,000132; 0,000319]	0,000631 [-0,000493; 0,00183]	0,0442 [0,0124; 0,0903]	0,000114 [6,3e-05; 0,000186]	0,00089 [0,000204; 0,00187]	0,000159 [2,62e-05; 0,000306]	0,00362 [-0,00291; 0,0122]
**Cult LPS- ALS**	0,001460 [0,001126; 0,002084], n=13	0,001183 [0,001083; 0,001538], n=13	0,01395 [0,009853; 0,01688], n=13	0,07322 [0,04129; 0,1557], n=13	0,0003509 [0,0002191; 0,0005079], n=13	0,006479 [0,004297; 0,007968], n=13	0,0007124 [0,0004659; 0,001068], n=13	0,01059 [0,004514; 0,03376], n=13

Compared PBMC subgroups	NLRP3	ASC	Procaspase-1	Pro-IL-1β	Pro-IL-18	IL-18BP	GSDMD	IL-6
**Cult LPS- HC**	0,001059 [0,0007338; 0,001240], n=10	0,001154 [0,0009314; 0,001289], n=10	0,01232 [0,008545; 0,01736], n=10	0,05117 [0,02643; 0,1062], n=10	0,0002939 [0,0002335; 0,0004238], n=10	0,004917 [0,003923; 0,006622], n=10	0,0006244 [0,0002545; 0,001106], n=10	0,009787 [0,003634; 0,01612], n=10
p-value (raw)	0,0120	0,3062	0,5981	0,2778	0,4025	0,3685	0,4025	0,3685
q (BH–FDR)	0,0961	0,4600	0,5981	0,4600	0,4600	0,4600	0,4600	0,4600
Effect size, r	+0,63	+0,26	+0,14	+0,28	+0,22	+0,23	+0,22	+0,23
Median diff [95% CI]	0,000449 [0,000333; 0,000674]	0,000177 [2,59e-05; 0,000254]	0,000813 [-0,000774; 0,00309]	0,0198 [0,00167; 0,0482]	5,33e-05 [-1,3e-05; 9,74e-05]	0,000767 [-8,47e-06; 0,0016]	0,000155 [-2,32e-05; 0,000319]	0,00362 [-9,1e-05; 0,0109]
**Cult LPS+ ALS**	0,001268 [0,001153; 0,002909], n=10	0,001113 [0,0009743; 0,001424], n=10	0,01067 [0,008664; 0,01276], n=10	0,3791 [0,1462; 0,8200], n=10	0,0005546 [0,0004780; 0,001238], n=10	0,002820 [0,002421; 0,007025], n=7	0,0008462 [0,0001974; 0,0009868[, n=7	0,07105 [0,04239; 0,08040], n=7
**Cult LPS+ HC**	0,001114 [0,0007593; 0,001554], n=5	0,0006717 [0,0006318; 0,0008963], n=5	0,01028 [0,008205; 0,01388], n=5	0,2973 [0,1127; 0,4932], n=5	0,0004310 [0,0002646; 0,0007617], n=5	0,002296 [0,002249; 0,003130], n=5	0,0003312 [0,0002611; 0,0008360], n=5	0,05646 [0,04642; 0,1229], n=5
p-value (raw)	0,0553	0,0400	0,9511	0,4396	0,2544	0,0480	0,7551	0,7551
q (BH–FDR)	0,1474	0,1474	0,9511	0,7033	0,5088	0,1474	0,8629	0,8629
Effect size, r	+0,64	+0,68	+0,04	+0,28	+0,40	+0,71	+0,14	-0,14
Median diff [95% CI]	0,000378 [0,000102; 0,000682]	0,000388 [0,000312; 0,000491]	0,000169 [-0,00164; 0,00195]	0,0856 [-0,032; 0,27]	0,000205 [5,49e-05; 0,000339]	0,000519 [0,000141; 0,00154]	0,000248 [-8,68e-05; 0,000521]	-0,00825 [-0,0342; 0,0146]
**Cult LPS+ (ALS and HC)**	0,001188 [0,001114; 0,001971], n=15	0,001016 [0,0006717; 0,001212], n=15	0,01062 [0,008669; 0,01263], n=15	0,3143 [0,1282; 0,6721], n=15	0,0005430 [0,0004310; 0,0008118], n=15	0,002579 [0,002308; 0,003891], n=12	0,0005849 [0,0002578; 0,0009531], n=12	0,06375 [0,04446; 0,08421], n=12
**Cult LPS- (ALS and HC)**	0,001197 [0,001054; 0,001728], n=23	0,001183 [0,0009811; 0,001428], n=23	0,01392 [0,008892; 0,01698], n=23	0,06121 [0,03228; 0,1210], n=23	0,0003221 [0,0002369; 0,0004630], n=23	0,005627 [0,004148; 0,007581], n=23	0,0007083 [0,0004106; 0,001069], n=23	0,009843 [0,004280; 0,02149], n=23
p-value (raw)	0,6542	0,0379	0,0731	0,0001	0,0021	0,0015	0,3480	<0,0001
q (BH–FDR)	0,6542	0,0607	0,0974	**0,0006**	**0,0042**	**0,0039**	0,3978	**0,0004**
Effect size, r	+0,09	-0,41	-0,35	+0,74	+0,60	-0,67	-0,20	+0,86
Median diff [95% CI]	6,01e-05 [-9,96e-05; 0,000145]	-0,000218 [-0,000332; -0,000127]	-0,00244 [-0,00396; -0,00105]	0,231 [0,131; 0,293]	0,000221 [0,00015; 0,000297]	-0,00213 [-0,00316; -0,00171]	-0,000138 [-0,000262; 4,95e-06]	0,0474 [0,0404; 0,0559]
**Cult LPS+ ALS**	0,001268 [0,001153; 0,002909], n=10	0,001113 [0,0009743; 0,001424], n=10	0,01067 [0,008664; 0,01276], n=10	0,3791 [0,1462; 0,8200], n=10	0,0005546 [0,0004780; 0,001238], n=10	0,002820 [0,002421; 0,007025], n=7	0,0008462 [0,0001974; 0,0009868[, n=7	0,07105 [0,04239; 0,08040], n=7
**Cult LPS- ALS**	0,001460 [0,001126; 0,002084], n=13	0,001183 [0,001083; 0,001538], n=13	0,01395 [0,009853; 0,01688], n=13	0,07322 [0,04129; 0,1557], n=13	0,0003509 [0,0002191; 0,0005079], n=13	0,006479 [0,004297; 0,007968], n=13	0,0007124 [0,0004659; 0,001068], n=13	0,01059 [0,004514; 0,03376], n=13
p-value (raw)	1,0000	0,2036	0,0824	0,0048	0,0084	0,0811	0,6992	0,0047
q (BH–FDR)	1,0000	0,2715	0,1318	**0,0191**	**0,0224**	0,1318	0,7991	**0,0191**
Effect size, r	+0,00	-0,32	-0,44	+0,71	+0,66	-0,49	-0,12	+0,76

Compared PBMC subgroups	NLRP3	ASC	Procaspase-1	Pro-IL-1β	Pro-IL-18	IL-18BP	GSDMD	IL-6
Median diff [95% CI]	-2,16e-05 [-0,000244; 0,000112]	-0,000136 [-0,000231; -3,87e-05]	-0,0032 [-0,00449; -0,00162]	0,273 [0,129; 0,351]	0,000243 [0,00014; 0,000345]	-0,00203 [-0,00366; -0,00118]	-0,000108 [-0,000262; 0,000138]	0,0432 [0,0307; 0,0578]
**Cult LPS+ HC**	0,001114 [0,0007593; 0,001554], n=5	0,0006717 [0,0006318; 0,0008963], n=5	0,01028 [0,008205; 0,01388], n=5	0,2973 [0,1127; 0,4932], n=5	0,0004310 [0,0002646; 0,0007617], n=5	0,002296 [0,002249; 0,003130], n=5	0,0003312 [0,0002611; 0,0008360], n=5	0,05646 [0,04642; 0,1229], n=5
**Cult LPS- HC**	0,001059 [0,0007338; 0,001240], n=10	0,001154 [0,0009314; 0,001289], n=10	0,01232 [0,008545; 0,01736], n=10	0,05117 [0,02643; 0,1062], n=10	0,0002939 [0,0002335; 0,0004238], n=10	0,004917 [0,003923; 0,006622], n=10	0,0006244 [0,0002545; 0,001106], n=10	0,009787 [0,003634; 0,01612], n=10
p-value (raw)	0,7679	0,0080	0,5135	0,0193	0,3097	0,0027	0,5941	0,0007
q (BH–FDR)	0,7679	**0,0213**	0,6789	**0,0386**	0,4955	**0,0107**	0,6789	**0,0053**
Effect size, r	+0,12	-0,84	-0,24	+0,76	+0,36	-0,92	-0,20	+1,00
Median diff [95% CI]	4,66e-05 [-0,000234; 0,000276]	-0,000349 [-0,000553; -0,000265]	-0,00145 [-0,00435; 0,000856]	0,219 [0,0763; 0,27]	0,00014 [1,19e-05; 0,000308]	-0,00193 [-0,00337; -0,00173]	-9,56e-05 [-0,000427; 5,78e-05]	0,048 [0,0409; 0,0824]

[1] – if "Cult" is not specified with "LPS- " or "LPS+ ", this indicates that LPS- and LPS+ PBMCs groups were merged and analyzed as one sample

Non-Cult – non-cultivated PBMCs

Cult – cultivated PBMCs

ALS– amyotrophic lateral sclerosis patients

HC – healthy controls

LPS- - LPS-untreated PBMCs

LPS+ - PBMCs treated with 1 μg/mL LPS for 3 hours

Supplementary Table S3. Statistical comparisons of relative protein expression levels of NLRP3 inflammasome components (NLRP3, ASC, pro-caspase-1) and pro-IL-1β. Protein expression levels were quantified by densitometry analysis (ImageJ software) of the Western blot data and were normalized to β-actin. The subgroups being compared are listed in the left column of the table, p-values were calculated by Mann-Whitney U tests, and for multiple testing, p-values were adjusted within each family of comparisons (the panel of proteins for a given contrast) with the Benjamini–Hochberg false-discovery-rate procedure (reported as q); p-values and an adjusted $q<0.05$ was considered statistically significant and are indicated in red; effect sizes are reported as the rank-biserial correlation; 95% confidence intervals for the difference in medians were estimated with the Hodges–Lehmann method. In the last section of the table, the data for ALS and HC groups were merged, as no statistically significant differences between ALS and HC were found.

Compared PBMC subgroups	NLRP3	ASC	Pro-caspase-1	Pro-IL-1β
**Non-Cult vs Cult**				
**Non-Cult ALS**	0,2684 [0,1298; 1,255] n=10	0,8820 [0,7607; 1,040] n=10	0,9413 [0,7991; 1,093] n=14	0,4614 [0,000; 0,6514], n=17
**Cult[1] ALS**	0,4770 [0,09582; 1,323] n=5	0,4291 [0,2405; 1,280] n=6	1,025 [0,8290; 1,921] n=14	0,8979 [0,4767; 1,464], n=16
p-value (raw)	1,0000	0,4278	0,2234	0,0058
q (BH–FDR)	1,0000	0,5704	0,4467	**0,0231**
Effect size, r	+0,00	+0,27	-0,28	-0,57
Median diff [95% CI]	0,00762 [-0,373; 0,188]	0,385 [-0,0514; 0,572]	-0,179 [-0,336; -0,0276]	-0,562 [-0,787; -0,395]
**Non-Cult HC**	0,4937 [0,06444; 1,369], n=9	0,6871 [0,5196; 0,8191], n=12	0,8768 [0,6865; 0,9296], n=15	0,3973 [0,02877; 0,7116], n=15
**Cult HC**	0,6201 [0,4514; 0,9473] n=4	0,6136 [0,2461; 1,355], n=8	0,9343 [0,6068; 1,617], n=12	1,421 [0,9479; 2,022], n=13
p-value (raw)	0,5886	0,6239	0,1960	<0,0001
q (BH–FDR)	0,6239	0,6239	0,3920	**0,0002**
Effect size, r	-0,22	+0,15	-0,30	-0,92
Median diff [95% CI]	-0,208 [-0,441; 0,142]	0,102 [-0,138; 0,259]	-0,182 [-0,462; -0,0367]	-1,02 [-1,29; -0,881]
**ALS vs HC**				
**Non-Cult ALS**	0,2684 [0,1298; 1,255], n=10	0,8820 [0,7607; 1,040], n=10	0,9413 [0,7991; 1,093], n=14	0,4614 [0,000; 0,6514], n=17
**Non-Cult HC**	0,4937 [0,06444; 1,369], n=9	0,6871 [0,5196; 0,8191], n=12	0,8768 [0,6865; 0,9296], n=15	0,3973 [0,02877; 0,7116], n=15
p-value (raw)	0,9025	0,1213	0,2475	0,7753
q (BH–FDR)	0,9025	0,4850	0,4949	0,9025
Effect size, r	-0,04	+0,40	+0,26	-0,06
Median diff [95% CI]	-0,0254 [-0,355; 0,139]	0,193 [0,0904; 0,264]	0,0997 [0,0221; 0,188]	-0,0179 [-0,14; 0,0362]
**Cult LPS- ALS**	0,4770 [0,09582; 1,323], n=5	0,4291 [0,2405; 1,280], n=6	1,025 [0,8290; 1,921], n=14	0,8979 [0,4767; 1,464], n=16
**Cult LPS- HC**	0,6201 [0,4514; 0,9473], n=4	0,6136 [0,2461; 1,355], n=8	0,9343 [0,6068; 1,617], n=12	1,421 [0,9479; 2,022], n=13
p-value (raw)	0,5556	0,9497	0,4253	0,1001

Compared PBMC subgroups	NLRP3	ASC	Pro-caspase-1	Pro-IL-1β
q (BH–FDR)	0,7407	0,9497	0,7407	0,4003
Effect size, r	-0,30	-0,04	+0,19	-0,37
Median diff [95% CI]	-0,269 [-0,4; 0,27]	-0,0421 [-0,359; 0,105]	0,178 [-0,0762; 0,364]	-0,47 [-0,766; -0,238]
**LPS- vs LPS+**				
**Cult LPS- (ALS and HC)**	0,4832 [0,2748; 0,8839], n=9	0,5286 [0,2555; 1,181] n=14	0,9964 [0,7870; 1,665] n=26	1,205 [0,5826; 1,716], n=29
**Cult LPS+ (ALS and HC)**	No data	0,6359 [0,2722; 0,7916], n=6	0,8061 [0,6439; 2,269] n=13	1,036 [0,6460; 2,427], n=14
p-value (raw)	No data	0,9044	0,5613	0,5254
q (BH–FDR)	No data	0,9044	0,8419	0,8419
Effect size, r		+0,05	+0,12	-0,12
Median diff [95% CI]	No data	0,0989 [-0,164; 0,322]	0,111 [-0,0713; 0,261]	-0,145 [-0,498; 0,15]

[1] – if “Cult” is not specified with “LPS- ” or “LPS+ ”, this indicates that LPS- and LPS+ PBMCs groups were merged and analyzed as one sample

Non-Cult – non-cultivated PBMCs

Cult – cultivated PBMC

LPS- - LPS-untreated PBMCs

LPS+ - PBMCs treated with 1 μg/mL LPS for 3 hours

ALS – amyotrophic lateral sclerosis patients

HC – healthy controls

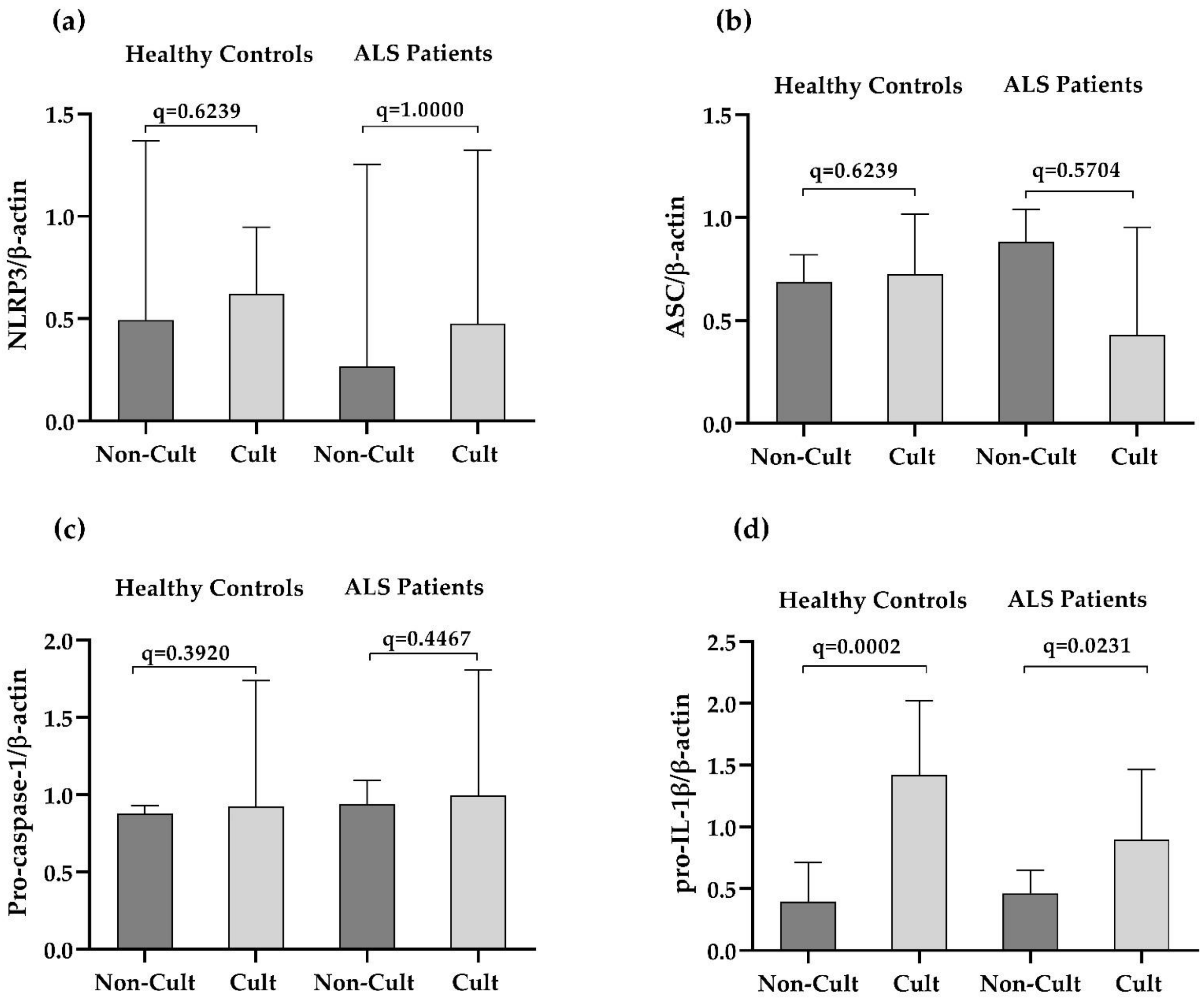
(a)
Healthy Controls
ALS Patients
q=0.6239
q=1.0000
NLRP3/β-actin
1.5
1.0
0.5
0.0
Non-Cult
Cult
Non-Cult
Cult
(b)
Healthy Controls
ALS Patients
q=0.6239
q=0.5704
ASC/β-actin
1.5
1.0
0.5
0.0
Non-Cult
Cult
Non-Cult
Cult
(c)
Healthy Controls
ALS Patients
q=0.3920
q=0.4467
Pro-caspase-1/β-actin
2.0
1.5
1.0
0.5
0.0
Non-Cult
Cult
Non-Cult
Cult
(d)
Healthy Controls
ALS Patients
q=0.0002
q=0.0231
pro-IL-1β/β-actin
2.5
2.0
1.5
1.0
0.5
0.0
Non-Cult
Cult
Non-Cult
Cult

Supplementary Figure S2. Protein expression levels of NLRP3 inflammasome components and IL-1β in non-cultivated and cultivated PBMCs of ALS patients and Healthy controls. The levels of NLRP3, ASC, pro-caspase-1 and pro-IL-1β proteins were analyzed in Western blotting of ALS and HC PBMCs, quantified by densitometry analysis (ImageJ) and normalized to β-actin signal. At least, 3 independent Western blot experiments were performed for each protein, with n=3-5 for each ALS, HC, Non-Cult and Cult group. Data are expressed as median with interquartile range, q values are shown on the graph, q<0,05 were considered significant, Mann-Whitney tests, with Benjamini–Hochberg false-discovery-rate procedure applied for multiple testing. The detailed data with statistical analysis including per group n values, effect sizes and 95% CI are represented in Supplementary Table 3. Non-Cult – non-cultivated PBMCs, Cult – cultivated PBMCs, ALS – amyotrophic lateral sclerosis patients, HC – healthy controls.

Supplementary Table S4. Statistical comparisons of IL-18 plasma concentration levels and IL-1β levels in cultivated PBMC supernatants. The subgroups being compared are listed in the left column of the table, p-values were calculated by Mann-Whitney U tests, and for multiple testing, p-values were adjusted within each family of comparisons (the panel of proteins for a given contrast) with the Benjamini–Hochberg false-discovery-rate procedure (reported as q); within-subject comparisons of LPS- vs LPS+ cultivated PBMCs, for which matched samples were available, were performed with the two-sided Wilcoxon signed-rank test. p-values and an adjusted q < 0.05 was considered statistically significant and are indicated in red; effect sizes are reported as the rank-biserial correlation; 95% confidence intervals for the difference in medians were estimated with the Hodges–Lehmann method.

Comparison	Group 1: median [Q1; Q3], n	Group 2: median [Q1; Q3], n	p-value	q (BH–FDR)	r	Median diff [95% CI]
**IL-18, plasma (pg/mL)**						
ALS vs HC *(Mann–Whitney U)*	ALS: 208,9 [157,7; 235,6], n=23	HC: 185,4 [168,8; 207,6], n=20	0,4147	–	+0,15	12,9 [-2,78; 28]
**IL-1β, cultivated PBMC culture supernatants (pg/mL)**						
ALS vs HC, LPS– *(Mann–Whitney U)*	ALS LPS–: 39,4 [11,0; 110,5], n=21	HC LPS–: 39,1 [22,7; 176,5], n=14	0,5891	0,5891	-0,11	-6,62 [-23,5; 7,54]
ALS vs HC, LPS+ *(Mann–Whitney U)*	ALS LPS+: 68,7 [46,6; 93,2], n=13	HC LPS+: 177,0 [93,0; 285,0], n=8	0,0638	0,1276	-0,50	-86,7 [-148; -34,7]
LPS– vs LPS+, ALS *(Wilcoxon (paired))*	ALS LPS–: 13,3 [0; 52,7], n=13	ALS LPS+: 68,7 [46,6; 93,2], n=13	0,0061	**0,0122**	+0,82	45,5 [28,4; 54,5]

Comparison	Group 1: median [Q1; Q3], n	Group 2: median [Q1; Q3], n	p-value	q (BH–FDR)	r	Median diff [95% CI]
LPS– vs LPS+, HC *(Wilcoxon (paired))*	HC LPS–: 47,8 [33,9; 138,4], n=8	HC LPS+: 177,0 [93,0; 285,0], n=8	0,0391	**0,0391**	+0,83	103 [65; 148]
